# Machine learning approach to evaluate TdP risk of drugs using cardiac electrophysiological model including inter-individual variability

**DOI:** 10.3389/fphys.2023.1266084

**Published:** 2023-10-04

**Authors:** Yunendah Nur Fuadah, Ali Ikhsanul Qauli, Aroli Marcellinus, Muhammad Adnan Pramudito, Ki Moo Lim

**Affiliations:** ^1^ Computational Medicine Lab, Department of IT Convergence Engineering, Kumoh National Institute of Technology, Gumi, Republic of Korea; ^2^ School of Electrical Engineering, Telkom University, Bandung, Indonesia; ^3^ Department of Engineering, Faculty of Advanced Technology and Multidiscipline, Universitas Airlangga, Surabaya, Jawa Timur, Indonesia; ^4^ Computational Medicine Lab, Department of Medical IT Convergence Engineering, Kumoh National Institute of Technology, Gumi, Republic of Korea; ^5^ Meta Heart Co., Ltd., Gumi, Republic of Korea

**Keywords:** torsade de pointes, drug risk assessment, in silico features, machine learning, grid search, explainable AI

## Abstract

**Introduction:** Predicting ventricular arrhythmia Torsade de Pointes (TdP) caused by drug-induced cardiotoxicity is essential in drug development. Several studies used single biomarkers such as qNet and Repolarization Abnormality (RA) in a single cardiac cell model to evaluate TdP risk. However, a single biomarker may not encompass the full range of factors contributing to TdP risk, leading to divergent TdP risk prediction outcomes, mainly when evaluated using unseen data. We addressed this issue by utilizing multi-*in silico* features from a population of human ventricular cell models that could capture a representation of the underlying mechanisms contributing to TdP risk to provide a more reliable assessment of drug-induced cardiotoxicity.

**Method:** We generated a virtual population of human ventricular cell models using a modified O’Hara-Rudy model, allowing inter-individual variation. ${\text{IC}}_{50}$ and Hill coefficients from 67 drugs were used as input to simulate drug effects on cardiac cells. Fourteen features ( ${\frac{\text{dVm}}{\text{dt}}}_{\text{repol}}$ , ${\frac{\text{dVm}}{\text{dt}}}_{\max}$ , ${\text{Vm}}_{\text{peak}}$ , ${\text{Vm}}_{\text{resting}}$ , ${\text{APD}}_{\text{tri}}$ , ${\text{APD}}_{90}$ , ${\text{APD}}_{50}$ , ${\text{Ca}}_{\text{peak}}$ , ${\text{Ca}}_{\text{diastole}}$ , ${\text{Ca}}_{\text{tri}}$ , ${\text{CaD}}_{90}$ , ${\text{CaD}}_{50}$ , qNet, qInward) could be generated from the simulation and used as input to several machine learning models, including k-nearest neighbor (KNN), Random Forest (RF), XGBoost, and Artificial Neural Networks (ANN). Optimization of the machine learning model was performed using a grid search to select the best parameter of the proposed model. We applied five-fold cross-validation while training the model with 42 drugs and evaluated the model’s performance with test data from 25 drugs.

**Result:** The proposed ANN model showed the highest performance in predicting the TdP risk of drugs by providing an accuracy of 0.923 (0.908–0.937), sensitivity of 0.926 (0.909–0.942), specificity of 0.921 (0.906–0.935), and AUC score of 0.964 (0.954–0.975).

**Discussion and conclusion:** According to the performance results, combining the electrophysiological model including inter-individual variation and optimization of machine learning showed good generalization ability when evaluated using the unseen dataset and produced a reliable drug-induced TdP risk prediction system.

## 1 Introduction

Torsades de Pointes (TdP) is a prevalent fatal arrhythmia symptom and a key indicator of sudden cardiac death events (Gintant, 2008; Frommeyer and Eckardt, 2016). Drug-induced TdP is one of the most common causes of drug withdrawal from the market (Gintant, 2008). Therefore, assessing drug-induced TdP is a critical issue in drug development. The International Council for Harmonization (ICH) has established guidelines (Cavero and Crumb, 2005) for assessing TdP risk caused by drugs. These guidelines, namely the S7B nonclinical evaluation and the E14 clinical evaluation guidelines, focus on two specific markers. One marker is the *in vitro* block of the hERG (human Ether-à-go-go-Related Gene) channel, which indicates the rapidly activating delayed rectifier potassium current (I_Kr_). The other marker is the prolongation of the QTc interval observed during clinical studies (FDA, 2005; EMEA, 2006). However, following these conventional guidelines necessitates extensive testing, leading to high sensitivity but low specificity in classifying drug risk (Colatsky et al., 2016). Consequently, even if the drugs do not present a Torsades de Pointes (TdP), they were subjected to strict regulations, revoked from the market, and dismissed in development (Llopis-Lorente et al., 2020). To address these issues, the FDA revised the guidelines for drug development by launching Comprehensive *in-vitro* Proarrhythmia Assay (CiPA) studies. Through *in silico* simulation, the CiPA group conducted the comprehensive evaluation of drug response in multiple ion channels, contrasting with a single assay evaluation that only uses the hERG channel (Crumb et al., 2016; Kun-Hee et al., 2018).

Several studies developed a drug testing system based on CiPA guidelines to classify TdP risk levels of drugs. Dutta et al. (2017) developed an *in silico* model based on the O’Hara-Rudy (ORD) human ventricular myocyte model (O'Hara et al., 2011). The proposed model by Dutta et al. (2017) optimized the ion channel maximal conductivities constant values of $I_{\text{Ks}}$ , $I_{\text{CaL}}$ , $I_{\text{Kr}}$ , $I_{\text{NaL}}$ , and $I_{K1}$ to 1.870, 1.007, 1.013, 2.661, and 1.698, respectively. Furthermore, it enabled evaluating the drug responses simulated *in silico* models similar to those obtained *in vitro*. They used qNet (the total amount of net charge transferred through 6 channels- $I_{\text{NaL}}$ , $I_{\text{CaL}}$ , $I_{\text{Kr}}$ , $I_{\text{to}}$ , $I_{K1}$ , and $I_{\text{Ks}}$ ) as features for classifying cardiotoxicity risk groups.

In addition to studies examining drug toxicity using single-cell models, several researchers attempted to evaluate TdP risk of drugs using 1D (line), 2D (tissue), and 3D (whole heart) models. As reported in review studies, Hwang et al. (2020) and Romero et al. (2018) analyzed the effects of 84 compounds on the QT interval by using pseudo-ECG from a 1D model. The authors proposed a novel torsagenic metric of a compound defined as the drug concentration yielding the 10% prolongation of APD and QT interval divided by the maximal effective free therapeutic concentration (EFTPCmax). Furthermore, research proposed by Polak et al. (2018) also utilized the pseudo-ECG from 1D simulation under 96 reference compounds to predict TdP risk of drugs in combination with several machine learning algorithms. The authors found that the decision tree was the best algorithm that could predict correctly 89% of reference drugs and 10 out of 12 validation drugs. In addition, studies using 2D simulations (Luo et al., 2017a; Luo et al., 2017b; Kubo et al., 2017) and 3D simulations (Hwang et al., 2019; Okada et al., 2015; Okada et al., 2018) examined simulated electrical wave propagation and ECG under the effects of various drugs to evaluate the TdP risk of drugs. However, despite promising results and findings from 1D, 2D, and 3D simulation studies, the analysis may require a substantial computational cost.

Li et al. (2019) proposed assessing the drug-induced TdP risk level into high-risk and low-risk using qNet as an input for a logistic regression model. This involved modifying the ORD model Dutta et al. (2017) proposed by adding hERG dynamics to generate qNet. Specifically, their research demonstrated that including hERG dynamic characteristics for classifying the TdP risk level of a drug improved the AUC compared with those not including hERG dynamic characteristics. The AUC of ROC1 (predicting the probability of low risk) was 0.901 and AUC of ROC2 (predicting the probability of high risk) was 0.988 when using the dynamic hERG model. In contrast, the AUC of ROC1 was 0.86 and AUC of ROC2 was 0.856 without the dynamic hERG model (Li et al., 2019). However, there are limited number of experimental data for dynamic hERG *in vitro* experiment and the data processing requires high computational complexity especially for dynamic hERG parameter estimation from *in vitro* data (Yoo et al., 2021).

The other studies proposed by Parikh et al. (2017) used the Early After Depolarization (EAD) metrics to evaluate drug-induced TdP risk. However, using EAD as a biomarker to predict TdP risk could be inferior to qNet metrics. EADs are very dependent on the ventricular cardiomyocyte model, which may have contributed to the poor performance, indicating the need to evaluate EADs using coupled cells or tissue models. Passini et al. (2017) used repolarization abnormalities (RAs) to indicate EAD. The prediction of TdP risk using RAs yielded 96% accuracy in simulation employing a population of 1,213 human ventricular control models with random ionic current changes. The simulation revealed that using RA in the virtual human population model provided a wider biological variety, leading to higher accuracy than a single model that only provided an accuracy of 59%.

Furthermore, Zhou et al. (2020) conducted blinded *in silico* drug trials using the optimized virtual human cell population proposed by Passini et al. to investigate the reliability of TdP risk prediction based on two independent sources. They used two datasets for evaluating the TdP risk prediction performance. Dataset I comprised 30 compounds, encompassing data on IC_50_ and Hill coefficients for seven distinct ionic currents: $I_{\text{Na}}$ , $I_{\text{NaL}}$ , $I_{\text{to}}$ (the transient outward potassium current), $I_{\text{Kr}}$ , $I_{\text{Ks}}$ , $I_{K1}$ (the inward rectifier potassium current), and $I_{Cal}$ (Crumb et al., 2016). On the other hand, Dataset II encompassed 55 compounds, yet it only contained data on IC_50_ and Hill coefficients for a subset of three ion channels: $I_{Na}$ , $I_{Kr}$ , and $I_{Cal}$ . The performance result obtained the highest accuracy of 83% using Dataset I and 80% using Dataset II. Their results confirmed that *in silico* simulations using an optimized population of human ventricular models are helpful tools for providing high-throughput TdP risk prediction.

Meanwhile, several researchers used multi-input features instead of a single biomarker to assess drug-induced TdP risk based on machine learning approaches. Polak et al. (2018) proposed a new methodology to estimate drug-induced TdP risk using ${\text{APD}}_{90}$ , ${\text{APD}}_{50}$ , pseudo-ECG signals, QRS width, QT interval, early repolarization time, and late repolarization time as *in silico* biomarkers feature extraction. Furthermore, they applied several machine learning algorithms: random forest, support vector machine (SVM), and decision tree. They reported the best classification accuracy of 89% using the empirical decision tree.

Parikh et al. (2017) used the inhibition rate of ion channels calculated through *in vitro* experiments as feature inputs into several classifier algorithms: logistic regression, support vector machines, and natural network model. Their study reported that the classification accuracy for each algorithm was 85%, 85%, and 86%, respectively. Meanwhile, Lancaster and Sobie. (2016) reported a high-performance AUC score of 0.962 using ${\text{APD}}_{50}$ and ${\text{Ca}}^{2+}$ as input of the SVM classification model. According to the simulation results, TdP risk was influenced by drug-induced changes to both the AP and intracellular ${\text{Ca}}^{2+}$ . Moreover, their study claimed that a measurement of ${\text{Ca}}^{2+}$ dynamics and the diastolic intracellular ${\text{Ca}}^{2+}$ provides the additional information necessary to classify the toxicity of drugs.

Furthermore, Yoo et al. (2021) used 28 of the drugs released by CiPA. They predicted their toxicity using nine *in silico* features ( ${\frac{\text{dVm}}{\text{dt}}}_{\max}$ , ${\text{APD}}_{\text{resting}}$ , ${\text{APD}}_{90}$ , ${\text{APD}}_{50}$ , ${\text{Ca}}_{\text{resting}}$ , ${\text{CaD}}_{90}$ , ${\text{CaD}}_{50}$ , qNet, qInward) as input to the ANN model. They obtained the highest AUC score of 0.92 for the high-risk group, 0.83 for the intermediate-risk group, and 0.98 for the low-risk group. According to the results, the ANN model performed well in classifying TdP risk. However, the model has not been validated using a different dataset with more compounds.

In the studies mentioned earlier using single-cell simulations, researchers commonly used the action potential morphology characteristics such as EADs based on repolarization abnormality (RA) or charge characteristics such as qNet from the ORD *in silico* model, which is highly correlated with the proarrhythmic risk level. Nevertheless, the univariate analysis using a single biomarker for TdP risk of drug assessment may not have sufficient generalization ability and lead to less robust predictions, such as in the study reported by Passini et al. (2017) using a single cardiac cell model that only provided an accuracy of 59% when using RA as a single biomarker.

Several studies proved that machine learning models could simultaneously leverage multiple biomarkers and other relevant features to make predictions. By considering a diverse range of information, they can capture complex relationships and interactions among variables, leading to improved predictive accuracy compared to relying on a single biomarker. However, the previous studies generated *in silico* features in a single cardiac model without considering inter-individual features that will be more reliable in evaluating the generalization ability of machine learning models. Moreover, the previous studies did not show the contribution of each feature to the performance of TdP risk assessment, which is very important for further analysis in drug development.

This study addresses unresolved issues in previous studies by combining the cardiac electrophysiological model including inter-individual variability and optimized machine learning models with grid search and explainable AI. We utilized 14 *in silico* features ( ${\frac{\text{dVm}}{\text{dt}}}_{\text{repol}}$ , ${\frac{\text{dVm}}{\text{dt}}}_{\max}$ , ${\text{Vm}}_{\text{peak}}$ , ${\text{Vm}}_{\text{resting}}$ , ${\text{APD}}_{\text{tri}}$ , ${\text{APD}}_{90}$ , ${\text{APD}}_{50}$ , ${\text{Ca}}_{\text{peak}}$ , ${\text{Ca}}_{\text{diastole}}$ , ${\text{Ca}}_{\text{tri}}$ , ${\text{CaD}}_{90}$ , ${\text{CaD}}_{50}$ , qNet, qInward) generated from the simulation of drugs effect in a population of human ventricular cardiac cell models as input to several machine learning models, including k-nearest neighbour (KNN), Random Forest (RF), XGBoost, and Artificial Neural Network (ANN). The optimization of the machine learning model was conducted using a grid search method for hyperparameter tuning automatically to provide the best parameters of the machine learning models. The models will be evaluated using the unseen dataset by analyzing evaluation metric performance, including accuracy, sensitivity, specificity, and AUC score. Furthermore, the contribution of each feature to the system performance will be demonstrated based on SHapley Additive exPlanations (SHAP) values of explainable AI (XAI). Therefore, the comprehensive approach in predicting the TdP risk of drugs based on *in silico* simulation with machine learning has the potential to be applied to drug development in the pharmaceutical industry.

## 2 Methods

**FIGURE 1:**
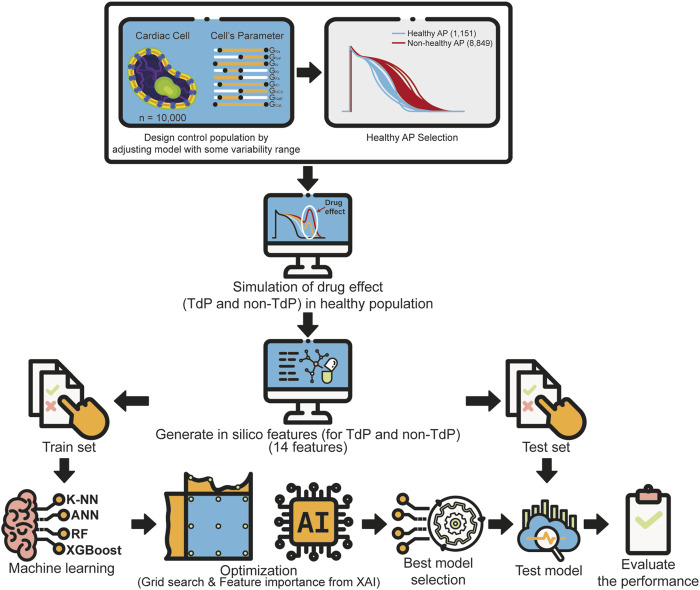
A general block diagram of the proposed method consisting of four main stages: design of the population of human ventricular cell models, *in silico* simulation to generate *in silico* features, drug’s TdP risk prediction using several machine learning models, and evaluation the system performance.

This study proposes a machine learning approach to evaluate drug-induced TdP risk based on a cardiac electrophysiological model including inter-individual variability to generate a control population of human ventricular cell models. The block diagram of the proposed method consisted of four main stages (Figure 1), which are the design of the population of human ventricular cell models, *in silico* simulation to generate *in silico* features, drug’s TdP risk prediction using several machine learning models, and evaluating the contribution of each feature to the prediction performance based on SHAP value of XAI.

### 2.1 Model of cardiac cell and drug’s effects

We used the O’Hara Rudy ventricular cell model modified by Dutta et al. (2017) to determine the drug’s effect on myocardial ionic channels. The membrane potential $\left(V_{m}\right.$ ) of myocardial cells can be calculated using the formula represented by Eq. 1.

$$\frac{\text{dVm}}{\text{dt}}=-\frac{1}{C_{m}}\left(I_{\text{total}}+I_{\text{stim}}\right)$$ (1)

where $I_{\text{total}}$ is the sum of transmembrane ionic currents that consist of sodium current ( $I_{\text{Na}}$ ), transient outward potassium current ( $I_{\text{to}}$ ), late sodium current ( $I_{\text{NaL}}$ ), L-type calcium current ( $I_{\text{CaL}}$ ), sodium current through L-type calcium channel ( $I_{\text{CaNa}}$ ), potassium current through L-type calcium channel ( $I_{\text{CaK}}$ ), rapid delayed rectifier potassium current ( $I_{\text{Kr}}$ ), slow delayed rectifier potassium current ( $I_{\text{Ks}}$ ), inward rectifier potassium current ( $I_{K1}$ ), sodium-calcium exchange current ( $I_{\text{NaCa}}$ ), sodium-potassium ATPase current ( $I_{\text{NaK}}$ ), background currents ( $I_{\text{Nab}}$ , $I_{\text{Cab}}$ , $I_{\text{Kb}}$ ), and sarcolemma calcium pump current ( $I_{\text{pCa}}$ ). Meanwhile, $I_{\text{stim}}$ is the current induced by an external stimulus. $C_{m}$ is the cell membrane capacitance set at 1.0μF for the experiment in this study (O'Hara et al., 2011).

We utilized the model of drug effects based on the study from Mirams et al. (2011) that was inspired by the work of Hill. (1910). The inhibition effects of the drug on the ion channel could be modeled through a conduction-block formulation as expressed by Eq. 2.

$$\text{inhibition effect}=\frac{1}{1+{\left(\frac{{\text{IC}}_{50}}{\left|D\right|}\right)}^{h}}$$ (2)

where the ${\text{IC}}_{50}$ represents the concentration of 50% inhibition of ionic current, $\left[D\right]$ represents the dosage of drugs, and h represents the Hill coefficient. The inhibition of the drug is assumed to affect multiple ion channels such as $C_{\text{aL}}$ , $K_{1}$ , $K_{s}$ , $N_{a}$ , $N_{\text{aL}}$ , $t_{o}$ , and $K_{r}$ or hERG. Finally, the ion channel’s conductance under the drug effect could be expressed as shown in Eq. 3.

$$g_{i}=g_{\text{control},i}\left(1-\text{inhibition effect}\right)$$ (3)

where the $g_{i}$ is the maximum conductance of ion channel i under drug effect and $g_{\text{control},i}$ is the maximum conductance of ion channel i without drug.

### 2.2 *In silico* simulation

**FIGURE 2:**
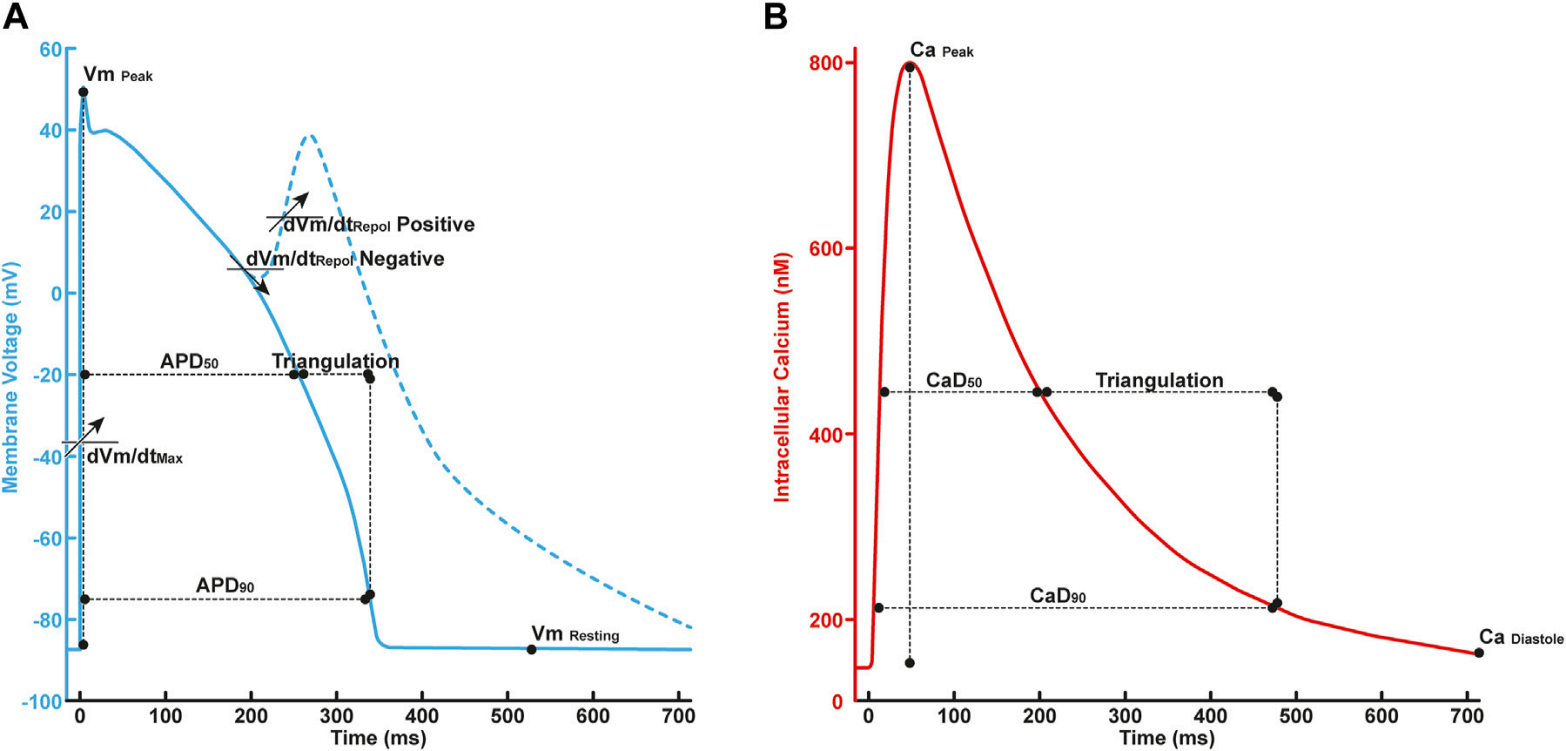
**(A)** The illustration of *in silico* features in AP profile consisted of ${APD}_{90}$ , ${APD}_{50}$ , ${APD}_{tri}$ , ${\frac{dVm}{dt}}_{repol}$ , ${\frac{dVm}{dt}}_{\max}$ , ${Vm}_{peak}$ , ${Vm}_{resting}$ ; **(B)** Ca profile consisted of ${CaD}_{90}$ , ${CaD}_{50}$ , ${Ca}_{tri}$ , ${Ca}_{peak}$ , ${Ca}_{diastole}$ .

*In silico* simulation of the drug’s effect was conducted to generate *in silico* features. The ${\text{IC}}_{50}$ and Hill coefficient were used in this study provided by Passini et al. (2017). The drug effects are simulated using various concentrations for each drug namely 1, 5, and 10. Initially, the cell’s voltage profile is simulated without adding drugs for 1,000 stimulations with a cycle length of 2,000 ms to reach the steady state condition. After that, drug effects were applied for 1,000 stimulations with the same cycle length. Following Chang et al. (2017a), the AP with the highest repolarization slope ( ${\frac{\text{dVm}}{\text{dt}}}_{\text{repol}}$ ) within the last 250 stimulations is selected to generate *in silico* features. The illustration for AP and Ca profiles is shown in Figure 2. For AP beat that fully repolarized, the search of ${\frac{\text{dVm}}{\text{dt}}}_{\text{repol}}$ is between 30%–90% repolarization; within 30% to the end of beat for AP beat that repolarises to 30% but not 90%, or between the peak of AP to the end of a cycle for AP beat that cannot repolarise by 30%. *In silico* features that we collected consist of the maximum rate of change of membrane potential during repolarization ( ${\frac{\text{dVm}}{\text{dt}}}_{\text{repol}}$ ), maximum membrane potential rate ( ${\frac{\text{dVm}}{\text{dt}}}_{\max}$ ), peak of action potential ( ${\text{Vm}}_{\text{peak}}$ ), resting value of membrane potential ( ${\text{Vm}}_{\text{resting}}$ ), action potential duration from peak to 90% and 50% repolarization ( ${\text{APD}}_{90}$ and ${\text{APD}}_{50}$ ), ${\text{APD}}_{\text{tri}}$ ( ${\text{APD}}_{90}$ - ${\text{APD}}_{50}$ ), peak of calcium intracellular concentration ( ${\text{Ca}}_{\text{peak}}$ ), diastolic intracellular calcium concentration ( ${\text{Ca}}_{\text{diastole}}$ ), calcium duration from peak to 90% and 50% repolarization ( ${\text{CaD}}_{90}$ and ${\text{CaD}}_{50}$ ), ${\text{Ca}}_{\text{tri}}$ ( ${\text{CaD}}_{90}$ - ${\text{CaD}}_{50}$ ), qNet, and qInward. Following Dutta et al. (2017), the qNet was defined as the total ionic charge during AP from six ion channels as shown in Eq. 4:

$$\text{qNet}=\int_{0}^{\text{BCL}}\left(I_{\text{Kr}}+I_{\text{CaL}}+I_{\text{to}}+I_{\text{NaL}}+I_{\text{Ks}}+I_{K1}\right)\text{dt}$$ (4)

Furthermore, the qInward, as studied by Li et al. (2019), was defined as shown in Eq. 5:

$$\text{qInward}=\frac{1}{2}\left(\frac{\int_{0}^{\text{BCL}}I_{\text{NaL},\text{drug}}\text{dt}}{\int_{0}^{\text{BCL}}I_{\text{NaL},\text{control}}\text{dt}}+\frac{\int_{0}^{\text{BCL}}I_{\text{CaL},\text{drug}}\text{dt}}{\int_{0}^{\text{BCL}}I_{\text{CaL},\text{control}}\text{dt}}\right)$$ (5)

### 2.3 Machine learning optimisation with grid search

Grid search is a commonly used technique in machine learning for optimizing hyperparameters. It systematically explores all possible combinations of hyperparameter values by creating a grid configuration (Elgeldawi et al., 2021; Gressling, 2021; Belete and Huchaiah, 2022). Each combination is trained and evaluated using a validation set to assess its performance. The goal is to identify the hyperparameter values that yield the best performance. While grid search effectively finds the best hyperparameters, it becomes inefficient when dealing with high-dimensional hyperparameter spaces. As hyperparameters and their potential values increase, the number of evaluations required grows exponentially. Specifically, if there are k parameters with n distinct values, the complexity of the grid search is expected to increase at a rate of $O_{\left(\text{nk}\right)}$ . To address this issue, it is essential to carefully constrain the hyperparameter search space to improve the efficiency of grid search as an optimization approach (Elgeldawi et al., 2021). Limiting the range of possible hyperparameter values can make the grid search process more focused and computationally manageable.

This study used four classifier algorithms: KNN, XGBoost, RF, and ANN. Our study’s selection of these classifier models was driven by their specific strengths and suitability for our research objectives (Supplementary Table S4 Supplementary Material). KNN is a non-parametric algorithm characterized by its ability to operate without making assumptions about the underlying data distribution (Sha’abani et al., 2020). It is particularly suitable for situations where the data distribution is not explicitly known or may exhibit non-standard characteristics. XGBoost employs a boosting technique to improve model performance sequentially by correcting errors (Hendrawan et al., 2022; Arif Ali et al., 2023). It has robustness in handling linear and non-linear relationships, including missing data (Hendrawan et al., 2022; Arif Ali et al., 2023). RF combines multiple decision trees to improve overall prediction accuracy and reduce overfitting (Belgiu and Drăguţ, 2016). Moreover, RF is less sensitive to outliers and noise in the data (Parmar et al., 2019). ANN has the advantage of exploring complex, non-linear patterns and hierarchical features in the dataset (Hamzah and Mohamed, 2020). ANN can be adapted to various problem domains through adjustments in architecture and hyperparameters (Hamzah and Mohamed, 2020; Tuan Hoang et al., 2021). By using these individual algorithms separately, we aimed to contrast their performance and applicability, offering a holistic evaluation of their suitability for predicting drug-induced TdP risk.

**FIGURE 3:**
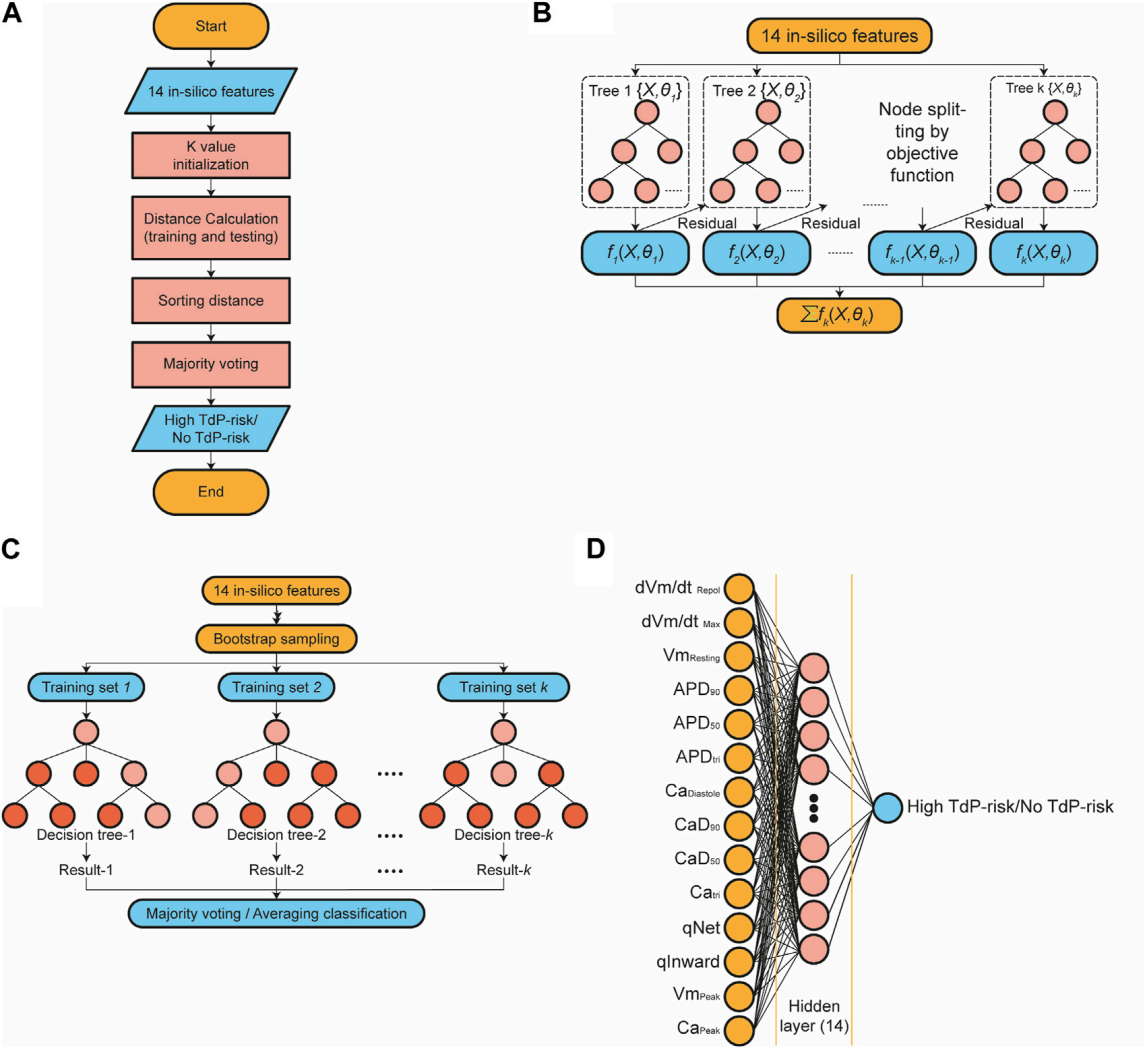
The schematic diagram of classifier models; **(A)** The diagram of the k-nearest neighbor classifier algorithm; **(B)** The topology of the XGBoost classifier algorithm; **(C)** The topology of the RF classifier algorithm. **(D)** The architecture of the artificial neural network algorithm.

Figure 3A presents the algorithm of the k-nearest neighbor (KNN) classifier algorithm employed in this study. In this approach, the training data underwent a projection into a multidimensional space, where each dimension denoted the *in silico* features obtained from the training data (Uddin et al., 2022; Fuadah et al., 2023). The training process encompassed the storage of feature vectors and associated labels. Meanwhile, during the prediction phase, the unlabeled testing data were labeled based on their proximity to the k nearest neighbors. Distances between feature vector positions in the training and testing data were computed using distance metrics within the multidimensional space, such as Euclidean, Chebyshev, and Minkowski. The prediction of the drug’s TdP risk is accomplished through majority voting based on the labels of the k-nearest neighbors. The optimization of the KNN model involved hyperparameter tuning utilizing the grid search method. The grid search method facilitated the selection of the best parameter values and the optimal k value from a range of options $\left(k=1,3,5,7,...,31\right)$ , as well as the appropriate distance metric, including Euclidean, Minkowski, and Chebyshev, throughout the optimization process.

The Extreme Gradient Boosting (XGBoost) classification algorithm is an enhanced method based on gradient-boosting decision trees, which efficiently constructs boosted trees and operates in parallel (Chen and Guestrin, 2016; Ibrahem Ahmed Osman et al., 2021; Montomoli et al., 2021; Tarwidi et al., 2023). Figure 3B illustrates a topology representation of the XGBoost classification process, where K represents the number of decision trees and $f_{k}X_{i}$ denotes the input function in the $k^{\text{th}}$ decision tree.

During training, the model continuously calculates node losses to identify leaf nodes with the most significant losses. XGBoost adds new decision trees by iteratively splitting input features. The objective of adding a new decision tree in XGBoost is to learn a new function, $f_{k}\left(X,\theta_{k}\right)$ , which complements the previous predictions. Once the training is completed and K decision trees are generated, each feature prediction sample corresponds to a leaf node in each decision tree, and each leaf node has an associated score. The scores from each tree are summed to obtain the final predicted value for that sample.

In this study, the XGBoost classification training model employs an ensemble of (50,100,150, and 200) decision trees. The complexity of the model increases with a higher number of decision trees. We set the options’ max depth parameter range (3, 5, 7, and 11). Additionally, the learning rate was also evaluated from 0.0001 to 0.1. The grid search method determined all the optimal tuning parameters used in this study.

The structure of the random forest (RF) classifier is shown in Figure 3C. RF comprises a group of decision tree classifier algorithms, which offer superior performance compared to using a single decision tree (Stavropoulos et al., 2020; Suhail et al., 2020; Xia, 2020). Random Forests combines two well-known classification tree approaches: boosting and bagging. It introduces an additional layer of randomness to the bagging technique. Both methods have distinct characteristics: boosting relies on the previous trees, assigning extra weight to misclassified points by earlier predictors, and making predictions based on weighted voting. On the other hand, bagging constructs each tree independently using a bootstrap sample of the dataset and makes predictions based on a simple majority vote.

RF incorporates two new strategies: Firstly, each tree is built using a different bootstrap sample of the data. Secondly, the splitting is performed at each node in the tree using the best predictor from a randomly selected subset of predictors rather than considering all variables as in standard trees. As a result, RF significantly modifies and improves upon the bagging approach by creating a diverse collection of uncorrelated trees and averaging their predictions.

In the classification process, all trees contribute by casting votes for their respective classes, and RF assigns the input to the class with the majority votes. The grid search technique identifies the optimal number of trees (50, 100, 150, and 200) and the best criterion (gini and entropy) that yields the highest performance outcome for the RF classifier.

An artificial neural network (ANN) is a fully connected architecture composed of three layers: input, hidden, and output layers (Shanbehzadeh et al., 2022; Pantic et al., 2023), as shown in Figure 3D. The input layer is responsible for receiving data from external sources. In this study, the input to the ANN architecture consisted of 14 *in silico* features. The hidden layers are responsible for processing the input from the preceding layer and transmitting the computed results to the output nodes. Specifically, the ANN utilized in this study incorporated one hidden layer comprising 14 nodes. The parametric rectified linear unit (PReLU) activation function was employed in the hidden layers, and a sigmoid activation function was utilized in the output layer to predict the TdP risk of drugs.

A grid search technique was employed to optimize the performance of the ANN model. The grid search aimed to identify the optimal choice of optimizer among Adam, Nadam, SGD, and RMSprop optimizers. Additionally, the grid search determined the optimal learning rate from 0.0001 to 0.1, yielding the highest performance for the ANN architecture.

### 2.4 Explainable AI for machine learning

Explainable AI, particularly in the context of machine learning, plays a crucial role in understanding the underlying factors driving predictions. In this study, we leveraged SHAP values to assess features’ importance in machine learning predictions. This approach is based on the concept of Shapley values from game theory, which was initially used to allocate rewards among players in a cooperative game (Lundberg et al., 2020). In the context of model interpretation, by calculating the SHAP values for each input feature, we gained insights into the contribution of individual features to the overall prediction.

In calculating SHAP values, the procedure initiates by establishing a baseline prediction, which is frequently determined by utilizing the model’s mean prediction over the entire dataset (Štrumbelj and Kononenko, 2014). The process involves systematically examining the impact of each feature by comparing the model’s prediction when including a particular feature and when excluding a particular feature. This difference reveals the extent to which a feature contributes to the prediction. Shapley values assign a credit to each feature based on its individual and collective impact on the prediction, ensuring that the contributions sum up correctly. Mathematically, the SHAP value ( ϕ ) for feature i on instance x is expressed as Eq. 6.

$$\emptyset_{i}\left(x\right)=\sum_{S\subseteq N\backslash \left\{i\right\}}\frac{\left|S\right|!\left(\left|N\right|-\left|S\right|-1\right)!}{\left|N\right|!}\left[f\left(x_{s}\cup \left\{i\right\}\right)-f\left(x_{s}\right)\right]$$ (6)

Where N is the set of features, S is a subset of N excluding feature i , $x_{s}$ is the instance x with only the features included in S set. Meanwhile, f is the model’s prediction function.

### 2.5 Evaluation of system performace

In measuring evaluation metrics including accuracy, sensitivity, and specificity, we have to measure the true positive (TP), the true negative (TN), the false positive (FP), and the false negative (FN). TP represents a situation in which the model correctly predicts the high TdP risk as a high TdP risk. TN represents a situation in which no TdP risk is predicted as no TdP risk (Sharma et al., 2022). FP is when no TdP risk is wrongly predicted as high TdP risk, while the FN is when high TdP risk is incorrectly predicted as no TdP risk. In addition, we calculated accuracies, sensitivity, and specificity using Eqs 7–9, respectively.

$$Accuracy=\frac{\text{TP}+\text{TN}}{\text{TP}+\text{TN}+\text{FP}+\text{FN}}$$ (7)

$$Sensitivity=\frac{\text{TP}}{\text{TP}+\text{FN}}$$ (8)

$$Specificity=\frac{\text{TN}}{\text{TN}+\text{FP}}$$ (9)

In addition, this research also reported the area under the curve (AUC) score to assesses the classifier’s ability in distinguish between different classes. The AUC scored obtained by measuring the area under Receiver Operating Characteristic (ROC) curve that plotted two metrics including true positive rate ( sensitivity ) and false positive rate ( $\left.1-Specificity\right)$ .

## 3 Result

### 3.1 Features generated from *in silico* simulations

In predicting the drug-induced TdP risk based on an electrophysiological model including inter-individual variability, we utilized 14 *in silico* features generated from *in silico* simulation of 67 drugs effect in 1,151 healthy control individuals. We provided the train and test set manually by adjusting 42 drugs as train set and 25 drugs as test set as shown in Table 1. The drugs were already categorized according to the TdP risk, which consists of 39 drugs of high TdP risk class and 28 drugs of no TdP risk class (Passini et al., 2017).

**TABLE 1:** The list of train and test drugs with EFPTCmax value.

Proarrhythmic risk level	Train drugs		Test drugs	
	Name	EFTPCmax (µM)	Name	EFTPCmax (µM)
High TdP-risk	Amiodarone I	0.155	Moxifloxacin I	10.96
	Amiodarone II	0.155	Moxifloxacin II	10.96
	Astemizole	0.0003	Moxifloxacin III	10.96
	Bepridil I	0.035	Pimozide	0.0005
	Bepridil II	0.035	Procainamide	54.18
	Bepridil III	0.035	Quinidine	3.237
	Chloropromazine I	0.038	Quinidine1	3.237
	Chloropromazine II	0.038	Sotalol I	14.69
	Cilostazol	0.128	Sotalol II	14.69
	Cisapride I	0.003	Sparfloxacin I	1.766
	Cisapride II	0.003	Sparfloxacin II	1.766
	Disopyramide	0.742	Terfenadine I	0.009
	Dofetilide I	0.0021	Terfenadine II	0.009
	Dofetilide II	0.0021	Terodiline	0.145
	Dofetilide III	0.0021	Thioridazine	0.98
	Donepezil	0.007		
	Droperidol	0.016		
	Flecainide I	0.752		
	Flecainide II	0.752		
	Flecainide III	0.752		
	Halofantrine	0.172		
	Haloperidol	0.004		
	Ibutilide	0.14		
	Methadone	0.507		
No TdP-risk	BaCl2	1	Nisoldipine	0.0001
	Ceftriaxone	23.17	Nitrendipine	0.003
	Diazepam	0.029	Pentobarbital	5.171
	Diltiazem I	0.1275	Phenytoin	4.36
	Diltiazem II	0.1275	Primidone	20.6
	Duloxetine	0.016	Piperacillin	114
	Lamivudine	19.54	Raltegravir	7
	Lidocaine I	2.6	Ribavirin	27.88
	Lidocaine II	2.6	Sitagliptin	0.442
	Linezolid	59.11	Telbivudine	19.72
	Loratadine	0.0004		
	Mexiletine I	2.5		
	Mexiletine II	2.5		
	Mibefradil I	0.012		
	Mibefradil II	0.012		
	Mitoxantrone	0.225		
	Nifedipine	0.008		
	Nimodipine	0.001		

Furthermore, we performed a correlation analysis between 14 *in silico* features to know which features highly correlated with one another. According to the correlation heatmap between features, as shown in Supplementary Figure S1 (Supplementary Material), the highest correlation value showed between ${\text{CaD}}_{50}$ and ${\text{CaD}}_{90}$ with a correlation value of 0.77, followed by ${\text{APD}}_{50}$ and ${\text{APD}}_{90}$ with a correlation of 0.75, and ${\text{Ca}}_{\text{diastole}}$ and ${\text{Vm}}_{\text{resting}}$ with a correlation value of 0.74. However, there are no features that have correlation values between one another greater than 0.8 that are commonly used as a threshold for feature selection (Cunningham et al., 2021; Taylor, 1990; Zampieri et al., 2008). Therefore, we used 14 *in silico* features for predicting drug-induced TdP risk. We applied Z-score normalization to preprocess these features before using them as input to the machine learning models (Al-Faiz et al., 2018; Raju et al., 2020).

**FIGURE 4:**
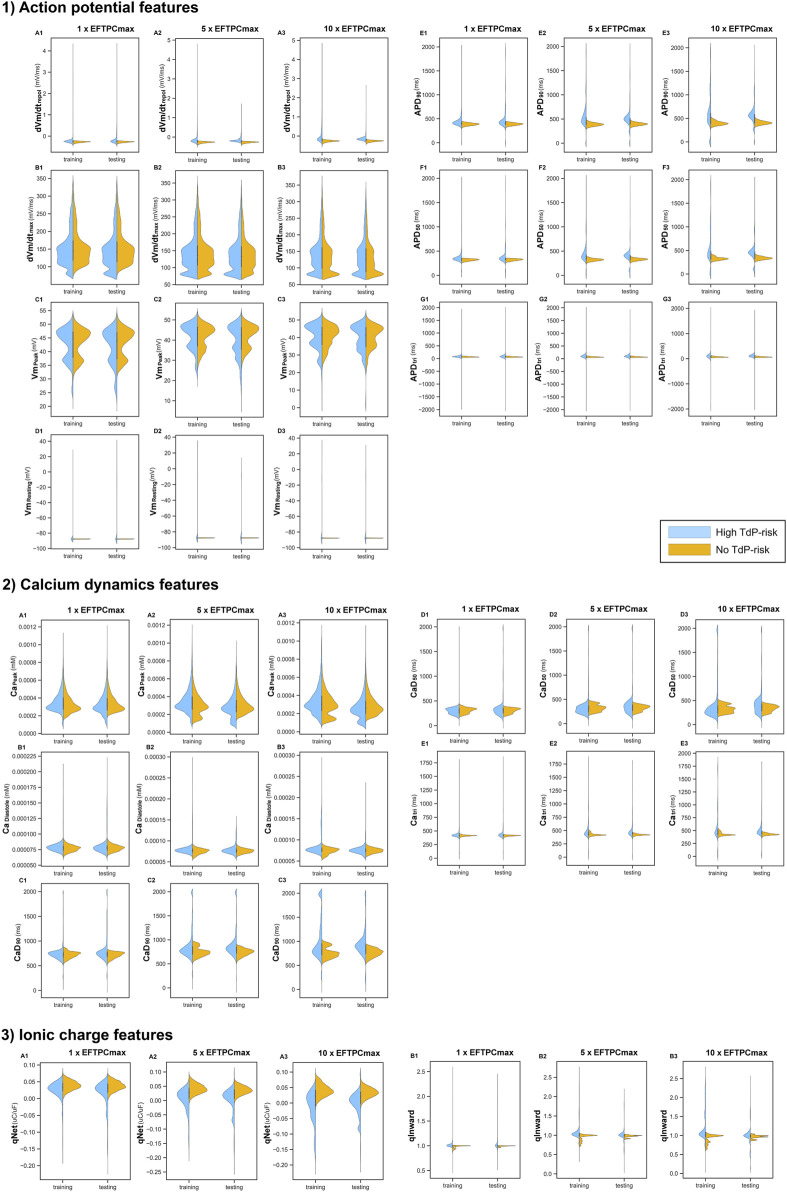
Features resulted from *in silico* simulations under 3 drug concentrations (1 × EFTPCmax, 5 × EFTPCmax, and 10 × EFTPCmax). Panel 1 represents the features obtained from AP profile, Panel 2 from calcium dynamics, and Panel 3 from ionic charge. The color represents the TdP-risk label of the drugs used in simulations.

Figure 4 shows the 14 features from *in silico* simulations under various drug concentrations. Some features varied mainly in a narrow region, with only a few samples filling a more comprehensive range of data. For example, from the AP features, only ${\frac{\text{dVm}}{\text{dt}}}_{\max}$ and ${\text{Vm}}_{\text{peak}}$ showed a relatively wider distribution of data than other features. Most features from AP shape had some outlier samples with much larger or smaller values than most of the data samples, making the distribution plot look narrower. Furthermore, from the calcium dynamics features, ${\text{Ca}}_{\text{tri}}$ features showed a considerably narrow data distribution with the majority of data samples distributed primarily on the range of ${\text{Ca}}_{\text{tri}}$ at 400–500 ms while some outlier samples produced values more than 1750 ms. In addition, from the ionic charge features, the qInward yielded a narrow data distribution, with the majority of the data samples located around 0.5–1.5, while the qNet resulted in a significantly wider data distribution compared to qInward.

The distribution of training and testing drugs showed changes when various drug concentrations were deployed. Some features such as ${\text{APD}}_{90}$ , ${\text{APD}}_{50},{\text{CaD}}_{90}$ , ${\text{CaD}}_{50}$ , ${\text{Ca}}_{\text{tri}}$ , and qNet yielded shifted, wider data distribution when higher drug concentrations were applied mainly for high TdP-risk drugs. Among these features, only qNet produced data distribution that shifted towards negative values, i.e., more samples produced smaller or negative qNet values when higher drug concentrations were administered. However, in contrast, the features generated under no TdP-risk drugs showed minimal changes when higher drug concentrations were applied.

### 3.2 Drugs induced TdP risk evaluation result

This study applied five-fold cross-validation with a grid search method to train 42 drugs using several machine learning models, including KNN, XGBoost, RF, and ANN. The grid search method provided the best hyperparameter setting to generate the best model of each machine learning model. Furthermore, the best model from each machine learning model was evaluated using the unseen dataset of 25 drugs, which consisted of 15 drugs of high TdP risk and 10 drugs of No TdP risk.

Our prediction models utilized simulations that observed the effect of drugs according to drug concentration variations at 1, 5, and 10 times EFTPCmax. Table 2 shows the performance in predicting drugs that induced TdP risk in 1, 5, and 10 × EFTPCmax concentrations using machine learning models. The highest performance for several machine learning models was provided at 10 × EFTPCmax. Among the several machine learning models in this study, the ANN model provided the best prediction of the drug’s TdP risk at 10 × EFTPCmax, followed by RF, XGBoost, and KNN.

**TABLE 2:** Drugs-induced TdP risk evaluation result with a 95% confidence interval according to EFTPCmax variation using several machine learning models.

Model	EFTPCmax	Accuracy	Sensitivity	Specificity	AUC
KNN	1 × EFTPCmax	0.862 (0.856–0.867)	0.867 (0.861–0.873)	0.853 (0.831–0.874)	0.86 (0.852–0.869)
	5 × EFTPCmax	0.876 (0.872–0.882)	0.873 (0.864–0.881)	0.882 (0.881–0.884)	0.878 (0.874–0.881)
	10 × EFTPCmax	0.875 (0.873–0.878)	0.87 (0.867–0.873)	0.884 (0.882–0.885)	0.876 (0.874–0.878)
XGBoost	1 × EFTPCmax	0.805 (0.804–0.806)	0.756 (0.719–0.793)	0.861 (0.845–0.877)	0.798 (0.788–0.808)
	5 × EFTPCmax	0.871 (0.868–0.874)	0.858 (0.850–0.865)	0.884 (0.878–0.889)	0.871 (0.870–0.871)
	10 × EFTPCmax	0.904 (0.901–0.907)	0.892 (0.886–0.899)	0.909 (0.903–0.914)	0.903 (0.902–0.905)
RF	1 × EFTPCmax	0.818 (0.817–0.819)	0.857 (0.856–0.857)	0.760 (0.759–0.762)	0.908 (0.907–0.908)
	5 × EFTPCmax	0.888 (0.887–0.889)	0.898 (0.897–0.899)	0.872 (0.871–0.875)	0.955 (0.954–0.956)
	10 × EFTPCmax	0.918 (0.917–0.919)	0.940 (0.939–0.941)	0.888 (0.887–0.888)	0.972 (0.971–0.973)
ANN	1 × EFTPCmax	0.827 (0.825–0.828)	0.885 (0.882–0.888)	0.788 (0.784–0.792)	0.889 (0.888–0.889)
	5 × EFTPCmax	0.899 (0.898–0.90)	0.938 (0.926–0.950)	0.873 (0.864–0.881)	0.947 (0.946–0.949)
	10 × EFTPCmax	**0.923 (0.908–0.937)**	**0.926 (0.909–0.942)**	**0.921 (0.906–0.935)**	**0.964 (0.954–0.975)**

The bold values mean the highest performance obtained.

We applied a grid search method for hyperparameter tuning automatically to select the best parameter of each classifier model. The grid search method selected the Adam optimizer with a learning rate of 0.001, 1,000 epochs, and a batch size 256 as the optimal parameter configuration for the ANN classifier model. Therefore, these parameters were employed to train the ANN model. Furthermore, for the RF model, the grid search selected entropy as the best criterion, with 100 trees as the optimal parameter of the RF model. For the XGBoost model, the grid search method determined 50 trees as the optimal number of estimators with three as maximum depth and learning rate 0.0001. Meanwhile, for the KNN algorithm, the grid search approach selected the Euclidean distance with a value of k = 1 as the best parameter for the KNN algorithm.

Furthermore, we have evaluated the model performance from each fold in five-fold cross-validation using the unseen dataset. The ANN model achieved the highest prediction performance with a 95% confidence interval when evaluated on the test data by obtaining an accuracy of 0.923 (0.908–0.937), sensitivity of 0.926 (0.909–0.942), specificity of 0.921 (0.906–0.935), and AUC score of 0.964 (0.954–0.975). The RF model prediction performance on test data obtained an accuracy of 0.918 (0.917–0.919), sensitivity of 0.940 (0.939–0.941), specificity of 0.888 (0.887–0.888), and AUC score of 0.972 (0.971–0.973).In addition, the XGboost model provided the highest performance on test data at 10 × EFTPCmax with an accuracy of 0.904 (0.901–0.907), sensitivity of 0.892 (0.886–0.899), specificity of 0.909 (0.903–0.914), and AUC score of 0.903 (0.902–0.905). Meanwhile, for the KNN algorithm, the highest classification performance at 5 × EFTPCmax. The best prediction obtained accuracy of 0.876 (0.872–0.882), sensitivity of 0.873 (0.864–0.881), specificity of 0.882 (0.881–0.884), and AUC score of 0.878 (0.874–0.881).

**FIGURE 5:**
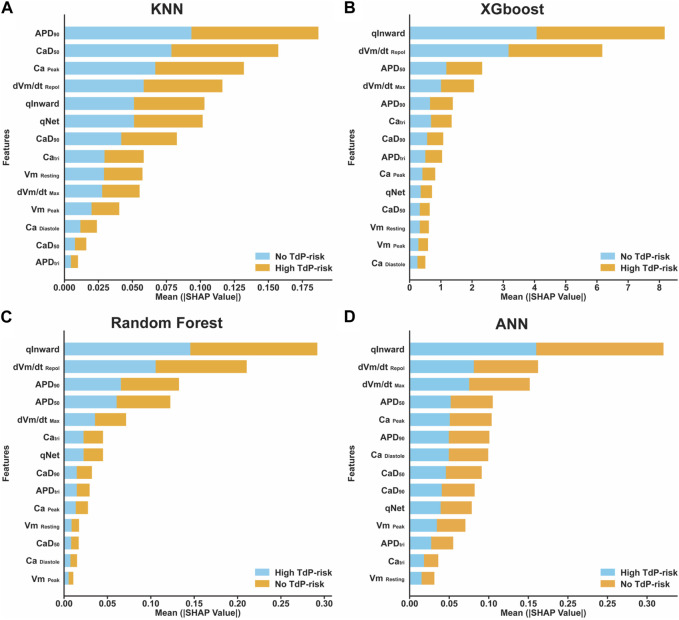
Features importance plot based on mean SHAP value in several classifier models. **(A)** The sum of mean SHAP value for KNN model; **(B)** The sum of mean SHAP value for XGBoost model; **(C)** The sum of mean SHAP value for RF model; and **(D)** The sum of mean SHAP value for ANN model.

We investigated a dataset with 14 features, including ${\frac{\text{dVm}}{\text{dt}}}_{\text{repol}}$ , ${\frac{\text{dVm}}{\text{dt}}}_{\max}$ , ${\text{Vm}}_{\text{peak}}$ , ${\text{Vm}}_{\text{resting}}$ , ${\text{APD}}_{\text{tri}}$ , ${\text{APD}}_{90}$ , ${\text{APD}}_{50}$ , ${\text{Ca}}_{\text{peak}}$ , ${\text{Ca}}_{\text{diastole}}$ , ${\text{Ca}}_{\text{tri}}$ , ${\text{CaD}}_{90}$ , ${\text{CaD}}_{50}$ , qNet, qInward. We used four classifier models: KNN, XGBoost, RF, and ANN, and conducted feature importance analysis using SHAP values to assess each feature’s contribution to the model’s predictions. As shown in Figure 5, the importance of features can vary depending on the classifier model. We observed some similarities and differences by comparing the feature importance across the different classifier models. For instance, qInward, ${\text{APD}}_{90}$ , ${\text{APD}}_{50}$ , and ${\frac{\text{dVm}}{\text{dt}}}_{\text{repol}}$ consistently emerged as essential features in all classifier models. Interestingly, certain features displayed varying levels of importance across the models. This disparity suggests that different classifier models emphasize different feature importance.

Initially, we examined the feature importance rankings provided by the SHAP values for each classifier model using all 14 features. Subsequently, we conducted additional experiments by removing certain features that did not exhibit significant contributions in the SHAP value analysis. We analyzed the contribution of each feature based on SHAP value for all machine learning models. We considered five different groups of features: the top 3, the top 6, the top 9, the top 11, and all 14 features as input to the classifier models. The top 3 features include qInward, ${\frac{\text{dVm}}{\text{dt}}}_{\text{repol}}$ , and ${\text{APD}}_{50}.$ The top 6 features include qInward, ${\frac{\text{dVm}}{\text{dt}}}_{\text{repol}}$ , ${\text{APD}}_{50}$ , ${\text{APD}}_{90},$ ${\frac{\text{dVm}}{\text{dt}}}_{\max},$ and ${\text{Ca}}_{\text{peak}}$ . The top 9 features include qInward, ${\frac{\text{dVm}}{\text{dt}}}_{\text{repol}}$ , ${\text{APD}}_{50}$ , ${\text{APD}}_{90},$ ${\frac{\text{dVm}}{\text{dt}}}_{\max},$ ${\text{Ca}}_{\text{peak}}$ , ${\text{Ca}}_{\text{tri}}$ , ${\text{CaD}}_{90},$ and qNet. The 11 features including qInward, ${\frac{dVm}{dt}}_{repol}$ , ${APD}_{50}$ , ${APD}_{90},$ ${\frac{dVm}{dt}}_{\max},$ ${Ca}_{peak}$ , ${Ca}_{tri}$ , ${CaD}_{90},$ qNet, ${CaD}_{90},$ and ${APD}_{tri}$ . However, our results indicated that removing certain features did not lead to significant differences in the performance of the classifier models. The models exhibited similar predictive performance and overall accuracy despite excluding features that did not demonstrate substantial contributions in the SHAP value analysis. This finding suggests that the excluded features may not have played a crucial role in the models’ decision-making processes. Table 3 compares the performance metrics achieved by the classifier models using the complete set of 14 features and the reduced sets of features. The metrics evaluated include accuracy, sensitivity, specificity, and AUC score with a 95% confidence interval. Notably, we observed that the performance of the ANN, RF, and XGBoost classifier models remained convergence for all feature groups with no statistically significant differences. Meanwhile, the KNN classifier model provided a smaller classification performance than other machine learning models.

**TABLE 3:** The comparison performance of machine learning models in predicting TdP risk of drug according to several features importance group based on mean SHAP value for each feature in classifier models.

Features	Model	Accuracy	Sensitivity	Specificity	AUC
3 Features	KNN	0.849 (0.848–0.849)	0.853 (0.852–0.853)	0.843 (0.842–0.844)	0.848 (0.847–0.848)
	XGBoost	0.884 (0.883–0.885)	0.897 (0.897–0.898)	0.874 (0.873–0.874)	0.886 (0.885–0.887)
	RF	0.896 (0.895–0.897)	0.899 (0.899–0.90)	0.891 (0.890–0.893)	0.958 (0.957–0.959)
	ANN	0.901 (0.898–0.903)	0.939 (0.937–0.940)	0.873 (0.870–0.875)	0.967 (0.966–0.968)
6 Features	KNN	0.853 (0.847–0.857)	0.876 (0.875–0.877)	0.819 (0.807–0.830)	0.848 (0.842–0.854)
	XGBoost	0.90 (0.897–0.903)	0.897 (0.884–0.910)	0.904 (0.877–0.930)	0.898 (0.894–0.902)
	RF	0.916 (0.915–0.916)	0.934 (0.933–0.935)	0.891 (0.889–0.892)	0.969 (0.968–0.969)
	ANN	0.913 (0.902–0.925)	0.921 (0.917–0.924)	0.908 (0.890–0.926)	0.96 (0.95–0.97)
9 Features	KNN	0.877 (0.876–0.880)	0.88 (0.878–0.882)	0.875 (0.873–0.877)	0.877 (0.875–0.879)
	XGBoost	0.90 (0.897–0.903)	0.897 (0.884–0.910)	0.904 (0.877–0.930)	0.898 (0.894–0.902)
	RF	0.917 (0.916–0.918)	0.937 (0.936–0.938)	0.889 (0.888–0.891)	0.970 (0.969–0.971)
	ANN	0.912 (0.91–0.913)	0.909 (0.899–0.919)	0.915 (0.909–0.922)	0.961 (0.959–0.964)
11 Features	KNN	0.879 (0.878–0.880)	0.881 (0.880–0.882)	0.876 (0.874–0.877)	0.878 (0.877–0.979)
	XGBoost	0.90 (0.889–0.912)	0.91 (0.899–0.923)	0.885 (0.879–0.891)	0.899 (0.887–0.908)
	RF	0.919 (0.918–0.920)	0.942 (0.941–0.943)	0.887 (0.885–0.889)	0.972 (0.971–0.972)
	ANN	0.913 (0.911–0.915)	0.903 (0.895–0.911)	0.920 (0.913–0.927)	0.963 (0.960–0.965)
14 Features	KNN	0.875 (0.873–0.878)	0.87 (0.867–0.873)	0.884 (0.882–0.885)	0.876 (0.874–0.878)
	XGBoost	0.904 (0.902–0.906)	0.912 (0.905–0.920)	0.893 (0.883–0.920)	0.903 (0.902–0.905)
	RF	0.918 (0.917–0.919)	0.940 (0.939–0.941)	0.888 (0.887–0.888)	0.972 (0.971–0.973)
	ANN1	**0.923 (0.908–0.937)**	**0.926 (0.909–0.942)**	**0.921 (0.906–0.935)**	**0.964 (0.954–0.975)**

The bold values mean the highest performance obtained.

As shown in Table 3, the ANN model provided the highest performance using 14 In silico features and still provided a good performance when only using 3 *in silico* features by providing an accuracy of 0.901 (0.898–0.903), sensitivity of 0.939 (0.937–0.940), specificity of 0.873 (0.870–0.875), and AUC score of 0.967 (0.966–0.968). However, removing several features in some cases leads to high sensitivity but low specificity or high specificity but low sensitivity. Based on the performance results using 3 *in silico* features, the higher sensitivity value compared to specificity value indicated that the model can predict high TdP risk, but is missclassified in predicting no TdP risk as high risk. Meanwhile, using 14 *in silico* features provided the highest sensitivity and specificity that show the model’s good capability in predicting both high TdP risk and no TdP risk.

Furthermore, we also evaluated the performance of machine learning models using the qNet feature proposed by Li et al. (2019) and ${\text{APD}}_{50}$ with ${\text{Ca}}_{\text{diastole}}$ proposed by Lancaster and Sobbie. (2016) The performance of machine learning models in predicting high and no TdP risk using qNet and ${\text{APD}}_{50}$ with ${\text{Ca}}_{\text{diastole}}$ is reported in Table 4. In predicting high and no TdP risk, all classifier models showed promissing results using ${\text{APD}}_{50}$ and ${\text{Ca}}_{\text{diastole}}$ as features with the highest performance provided by ANN model with an accuracy of 0.890 (0.882–0.897), sensitivity of 0.895 (0.887–0.902), specificity of 0.886 (0.879–0.893), and AUC score of 0.951 (0.944–0.957). Nevertheless, the performance decreased when only using qNet feature. The XGBoost and RF, model showed the similar classification performance as the ANN model that obtained an accuracy of 0.762 (0.761–0.763), sensitivity of 0.761 (0.760–0.762), specificity of 0.763 (0.762–0.764), and AUC score of 0.843 (0.842–0.844). In contrast, the KNN model obtained the lowest performance compared to others classifier models. This shows that using univariate feature could not capture the complete discriminatory factors contributing to the TdP risk of drugs compared to utilizing multiple *in silico* features.

**TABLE 4:** The comparison performance of machine learning models using qNet feature, ${\text{APD}}_{50}$ & ${\text{Ca}}_{\text{diastole}}$ features, and 14 *in silico* features in predicting high and no TdP risk of drugs.

Model	Features	Accuracy	Sensitivity	Specificity	AUC
KNN	qNet	0.544 (0.538–0.549)	0.220 (0.210–0.239)	0.998 (0.997–0.998)	0.609 (0.605–0.615)
	${\text{APD}}_{50}$ & ${\text{Ca}}_{\text{diastole}}$	0.875 (0.873–0.878)	0.870 (0.867–0.873)	0.884 (0.882–0.885)	0.876 (0.874–0.878)
	14 In Silico Features	0.896 (0.895–0.897)	0.899 (0.898–0.90)	0.891 (0.890–0.893)	0.958 (0.957–0.959)
XGBoost	qNet	0.761 (0.760–0.762)	0.773 (0.763–0.783)	0.754 (0.744–0.764)	0.763 (0.762–0.764)
	${\text{APD}}_{50}$ & ${\text{Ca}}_{\text{diastole}}$	0.850 (0.848–0.852)	0.823 (0.796–0.850)	0.864 (0.831–0.897)	0.844 (0.842–0.846)
	14 In Silico Features	0.904 (0.902–0.906)	0.912 (0.905–0.920)	0.893 (0.883–0.920)	0.903 (0.902–0.904)
RF	qNet	0.762 (0.761–0.763)	0.752 (0.748–0.757)	0.775 (0.768–0.781)	0.843 (0.842–0.844)
	${\text{APD}}_{50}$ & ${\text{Ca}}_{\text{diastole}}$	0.831 (0.830–0.832)	0.873 (0.871–0.875)	0.772 (0.771–0.773)	0.911 (0.910–0.912)
	14 In Silico Features	0.918 (0.917–0.919)	0.940 (0.939–0.941)	0.888 (0.887–0.889)	0.972 (0.971–0.973)
ANN	qNet	0.762 (0.761–0.763)	0.761 (0.760–0.762)	0.763 (0.762–0.764)	0.843 (0.842–0.844)
	${\text{APD}}_{50}$ & ${\text{Ca}}_{\text{diastole}}$	0.890 (0.882–0.897)	0.895 (0.887–0.902)	0.886 (0.879–0.893)	0.951 (0.944–0.957)
	14 In Silico Features	**0.923 (0.908–0.937)**	**0.926 (0.909–0.942)**	**0.921 (0.906–0.935)**	**0.964 (0.954–0.975)**

The bold values mean the highest performance obtained.

According to the performance results, the proposed method using 14 *in silico* features with machine learning models obtained the highest performance in predicting high and no TdP risk of drugs. For further analysis, since CiPA categorized the TdP risk level of the drug into three categories, we also evaluated the performance of the proposed method in predicting high, intermediate, and low TdP risk as shown in Table 5. However, the proposed method could not optimally predict the TdP risk of drugs, especially due to the imbalanced class of the dataset between the high, intermediate, and low-risk categories of drugs (Supplementary Table S3. Supplementary Material).

**TABLE 5:** The comparison performance of machine learning models using qNet feature, ${\text{APD}}_{50}$ & ${\text{Ca}}_{\text{diastole}}$ features, and 14 *in silico* features in predicting high, intermediate, and no TdP risk of drugs.

Model	Features	Accuracy	Sensitivity	Specificity	AUC
KNN	qNet	0.608 (0.606–0.609)	0.403 (0.401–0.406)	0.702 (0.701–0.704)	0.562 (0.559–0.565)
	${\text{APD}}_{50}$ & ${\text{Ca}}_{\text{diastole}}$	0.747 (0.741–0.753)	0.582 (0.576–0.588)	0.809 (0.806–0.812)	0.758 (0.746–0.769)
	14 In Silico Features	0.762 (0.761–0.763)	0.607 (0.606–0.608)	0.816 (0.815–0.817)	0.711 (0.710–0.712)
XGBoost	qNet	0.718 (0.717–0.719)	0.515 (0.514–0.516)	0.775 (0.774–0.776)	0.702 (0.701–0.703)
	${\text{APD}}_{50}$ & ${\text{Ca}}_{\text{diastole}}$	0.789 (0.784–0.796)	0.627 (0.621–0.632)	0.804 (0.834–0.841)	0.804 (0.799–0.809)
	14 In Silico Features	0.798 (0.785–0.810)	0.650 (0.620–0.679)	0.841 (0.829–0.854)	0.883 (0.867–0.899)
RF	qNet	0.718 (0.717–0.719)	0.515 (0.514–0.516)	0.775 (0.774–0.776)	0.703 (0.702–0.70)
	${\text{APD}}_{50}$ & ${\text{Ca}}_{\text{diastole}}$	0.790 (0.789–0.791)	0.616 (0.615–0.617)	0.834 (0.833–0.835)	0.794 (0.793–0.795)
	14 In Silico Features	0.835 (0.831–0.839)	0.705 (0.697–0.714)	0.869 (0.866–0.872)	0.905 (0.904–0.906)
ANN	qNet	0.719 (0.718–0.720)	0.517 (0.515–0.519)	0.776 (0.775–0.777)	0.705 (0.704–0.706)
	${\text{APD}}_{50}$ & ${\text{Ca}}_{\text{diastole}}$	0.790 (0.788–0.791)	0.620 (0.616–0.625)	0.835 (0.833–0.836)	0.801 (0.799–0.803)
	14 In Silico Features	**0.852 (0.846–0.857)**	**0.762 (0.758–0.766)**	**0.888 (0.886–0.889)**	**0.911 (0.907–0.914)**

The bold values mean the highest performance obtained.

The classification performance of three classes TdP risk of drugs provided the highest performance using 14 *in silico* features as input to the classifier models. The ANN model obtained an accuracy of 0.852 (0.846–0.857), sensitivity of 0.762 (0.758–0.766), specificity of 0.888 (0.886–0.889), and AUC score of 0.911 (0.907–0.914). The low sensitivity and high specificity indicated the model misclassified high and intermediate TdP risk as no TdP risk but most of no TdP risk classified correctly as no TdP risk. Therefore, for the overall performance, the model provided high accuracy and AUC score that showed the good capabilities of the model in differentiating between classes.

Furthermore, the RF model provided an accuracy of 0.835 (0.831–0.839), sensitivity of 0.705 (0.697–0.714), specificity of 0.869 (0.866–0.872), and AUC score of 0.905 (0.904–0.906). The XGBoost model provided the classification performance with an accuracy of 0.798 (0.785–0.810), sensitivity of 0.650 (0.620–0.679), specificity of 0.841 (0.829–0.854), and AUC score of 0.883 (0.867–0.899). Meanwhile, the KNN model obtained the lowest classification performance with an accuracy of 0.762 (0.761–0.763), sensitivity of 0.607 (0.606–0.608), specificity of 0.816 (0.815–0.817), and AUC score of 0.711 (0.710–0.712). In contrast with using 14 *in silico* features as input to the machine learning models for multiclass classification, utilizing qNet, or ${\text{APD}}_{50}$ with ${\text{Ca}}_{\text{diastole}}$ as features lead to decreasing classification performance of high, intermediate, and low TdP risk of drugs. It became evident that using only one or two features was insufficient in capturing the comprehensive set of factors contributing to the TdP risk of drugs when contrasted with the utilization of all 14 *in silico* features.

## 4 Discussion

The findings of this study contribute to the ongoing efforts to improve the prediction of drug-induced TdP risk by combining an electrophysiological model including inter-individual variability with optimized machine learning algorithms. The results demonstrate the potential of utilizing 14 *in silico* features derived from a human ventricular cardiac cell model population to accurately predict TdP risk using several machine learning models.

Previous studies have primarily relied on single *in silico* biomarkers such as qNet and Repolarization Abnormality (RA) in a single cardiac cell model. These biomarkers have shown a high correlation with TdP risk. However, their prediction ability may be limited because single biomarkers only encompass part of the factors contributing to TdP risk. Analysis with a single *in silico* biomarker as the input feature could result in feature thresholds to differentiate TdP risk of drugs like the one proposed by (Li et al., 2019) that suggested qNet as the TdP metric. By incorporating multiple *in silico* features, our approach could capture a representation of the underlying mechanisms contributing to TdP risk, and a more comprehensive assessment of cardiac electrophysiology can be achieved. Furthermore, since the classification of TdP risk of drugs using the proposed machine learning algorithm considered multiple inputs and complex machine learning structure, the model did not consider feature thresholds as the discriminant of TdP risk of drugs.

In addition, single biomarkers may fail to capture the interactions between different factors. Cardiac electrophysiology is a highly interconnected system where alterations in one aspect can affect numerous others. Considering only a single biomarker, the interdependencies and complex relationships between different biomarkers need to be adequately addressed. This limitation can result in an oversimplified view of drug-induced TdP risk, potentially leading to inaccurate predictions.

Furthermore, this study incorporates inter-individual variability through a population of human ventricular models. The study proposed by Passini et al. (2017) that used a population of human ventricular models for 49 drugs reported an accuracy of 96%, sensitivity of 100%, and specificity of 92% using RAs in calculating the TdP score. Meanwhile, using ${APD}_{90}$ in calculating the TdP score, the prediction performance obtained an accuracy of 80%, sensitivity of 96%, and specificity of 64%. The virtual human population model resulted in a broader range of biological variations, leading to superior accuracy compared to a single model. However, their proposed model still needed to evaluate the proposed approach using the unseen dataset to evaluate the robustness and generalization ability of the proposed method.

Zhou et al. (2020) performed blinded *in silico* drug trials, employing the optimized virtual human cell population proposed by Passini et al. (2017) to assess the dependability of the drug’s TdP risk prediction from two different sets of drugs. The highest accuracy achieved was 83% with dataset I and 80% with dataset II. These results substantiate the effectiveness of *in silico* simulations utilizing an optimized population of human ventricular models as valuable resources for facilitating high-throughput TdP risk prediction. Therefore, we adopted their approach by incorporating inter-individual variability in generating a control population of the human ventricular model. We combined the electrophysiological model with several machine learning models to improve drug-induced TdP risk prediction performance, especially when evaluating the unseen dataset.

The study highlights the significance of considering drug concentration (EFTPCmax) in predicting TdP risk. As shown in Table 2, various drug concentrations affected the prediction of each machine-learning model. The rationale behind using different variations of drug concentration, which are 1 × EFTPCmax, 5 × EFTPCmax, and 10 × EFTPCmax for predicting drug-induced TdP risk using a machine learning model lies in the consideration of different potential impact on drug-induced cardiac effects. Based on the prediction performance under several drug concentrations, in general, 10 × EFTPCmax provided the highest performance compared to 5 × EFTPCmax and 1 × EFTPCmax for most machine learning models. At 10 × EFTPCmax, the prediction performance of the machine learning model, especially the ANN, provided the highest performance with no significant gap for sensitivity and specificity. These results indicated that at 10 × EFTPCmax provided balanced performance, reliable prediction and good generalization ability of unseen datasets in predicting high TdP and no TdP risk.

Meanwhile, the performance of machine learning prediction at 5 × EFTPCmax and 1 × EFTPCmax mostly leads to high sensitivity but low specificity, which indicates the model is more accurate in predicting high TdP risk instead of no TdP risk. In addition, several results at 5 × EFTPCmax and 1 × EFTPCmax show high specificity but low sensitivity, indicating that the model is more accurate in predicting no TdP risk than high TdP risk. Therefore, we used 10 × EFTPCmax for analysis of the contribution of each feature based on SHAP value of XAI.

The previous studies conducted by Passini et al. (2017) and Zhou et al. (2020) have predominantly relied on the biomarker 100 × EFTPCmax for predicting TdP risk. However, in this study, we observed that good prediction performance could be achieved by considering the biomarker at a lower value of 10 × EFTPCmax. The result is in line with the findings of Passini et al. (2017), which also demonstrates the advantage of considering a lower biomarker value, specifically at 10 × EFTPCmax, in predicting TdP risk. Considering a lower biomarker value can capture relevant information at an earlier stage of drug exposure, potentially enabling the identification of TdP risk at an earlier time. This early detection is crucial for timely intervention and preventing adverse cardiac events.

The comparison of different machine learning models revealed that the ANN model best predicted TdP risk of the unseen dataset at 10 × EFTPCmax. The prediction performance obtained an accuracy of 0.923 (0.908–0.937), sensitivity of 0.926 (0.909–0.942), specificity of 0.921 (0.906–0.935), and AUC score of 0.964 (0.954–0.975). This finding suggests that the ANN model effectively captures the complex relationships between the *in silico* features and TdP risk, leading to more accurate predictions. The ANN model provided high sensitivity and specificity, indicating the proposed model’s ability to predict high TdP and no TdP risk. It is also supported by the high AUC score that indicates the model has a high true positive rate (sensitivity) and a low false positive rate (1-specificity). The highest AUC score indicates a robust and reliable predictive model for distinguishing between high TdP and no TdP instances.

In this study, we have also evaluated the classification performance of machine learning to predict high, intermediate, and no TdP risk groups. The classification performance for binary and three-class classification analyses showed valuable insights into the predictive capabilities of machine learning models in assessing TdP risk among drugs. The ANN model exhibited commendable performance in binary classification, with ANN classification performance outperforming the other classifier performance. These models demonstrated high accuracy, sensitivity, specificity, and AUC scores, underscoring their efficacy in distinguishing between high and no TdP risk drugs. Conversely, for more complex three-class classification, the ANN model also provides comparable performance of accuracy and AUC scores, indicating its potential for categorizing compounds into high, intermediate, and low TdP risk groups. However, the challenge of imbalanced classes affected sensitivity, particularly in the intermediate risk category.

RF and XGBoost exhibited good performance for predicting high and no TdP risk, but their ability to identify drug risk categories correctly decreased for classifying high, intermediate, and no TdP risk. On the other hand, KNN, which performed reasonably well in binary classification, showed a decline in its performance when dealing with multiple risk categories. These results underscore the need for specialized approaches to address the complexities of multi-class classification and emphasize the promise of advanced machine learning models in enhancing drug-induced TdP risk assessments.

In addition, this study also applied XAI to show the contribution of each feature to predict drug-induced TdP risk based on SHAP value. The results from feature importance in Figure 5 showed that qInward, ${APD}_{90}$ , ${APD}_{50}$ , and ${\frac{dVm}{dt}}_{repol}$ were the important features for classifying drug toxicity. The results were in alignment with previous studies (Obiol-Pardo et al., 2011; Chang et al., 2017b; Romero et al., 2018; Llopis-Lorente et al., 2020; Jeong, Qashri Mahardika et al., 2022). Jeong et al. (2022) examined the qInward variability and showed that it was superior to other *in silico* features for classifying the TdP risk of drugs. Moreover, Romero et al. (2018) and Llopis-Lorente et al. (2020) showed other features related to APD, such as the ratio of drug concentrations leading to 10% prolongation of ${APD}_{90}$ , (Tx-APD) over the EFTPCmax showed good performance of classifying torsadogenic compounds. In addition, the study from Chang et al. (2017b) utilized the ${\frac{dVm}{dt}}_{repol}$ feature to filter the AP beat for calculating the *in silico* features. The highest ${\frac{dVm}{dt}}_{repol}$ indicated the most affected AP beat by the drug that could cause AP prolongation or even EAD. Therefore it could be possible that ${\frac{dVm}{dt}}_{repol}$ became an important feature for classifying the TdP risk of drugs.

This study addresses several limitations of previous research by considering inter-individual variability through a population of human ventricular models. The current approach reflects the human population’s heterogeneity and improves the predictions’ generalizability. Additionally, using optimized machine learning models through grid search hyperparameter tuning enhances the accuracy and robustness of the predictions. Moreover, the SHAP value based on XAI showed the contribution of each feature to the prediction performance. Our findings emphasize the need to consider carefully the feature set in machine learning studies. By selecting an appropriate subset of features, we can achieve comparable model performance while reducing the dimensionality of the data.

Albeit the promising results shown in this study, some limitations must be considered. First, this study only examined one cardiac cell model, whereas another cellular model, such as the one proposed by Tomek et al. (2019) could also be used for cardiac drug toxicity evaluation. According to this model, the ORD model inherits several inconsistencies when compared to experimental data, including higher AP than experimental data during the plateau stage, limited agreement to experimental observation for the dynamics of accommodation of the APD to heart rate acceleration, and simulation results of sodium current block that demonstrate an inotropic effect that increases the calcium transient amplitude. To address several ORD model limitations, Tomek et al. (2019) suggested some changes, namely in reformulating ICaL and reevaluating IKr. Imposing several cell models into *in silico* simulation might provide more insight into the reliability of the machine learning model to predict the TdP risk of compounds. Furthermore, the drugs reported a high performance in predicting high and no TdP risk. However, the performance for three class classification (high, intermediate, and no TdP risk of drugs) still needs improvement. Therefore, incorporating more drug datasets in the training and testing stage for machine learning models to perform multiclass classification could be an essential step in future research.

Another possible approach is the multiscale drug toxicity evaluation simulation that incorporates whole-heart simulations to predict a more realistic outcome. However, incorporating the drug effects of several drug samples on various individuals (by imposing the inter-individual variability mechanism) into whole-heart simulation may require a significantly higher computational cost tha single-cell simulations. For example, one whole heart simulation may consist of hundred of thousands of computational nodes (or cells) and millions of elements, such as the one utilized by Qauli et al. (2022) to verify the efficacy of the mexiletine for treatment of patients with A1656D mutation or the one used by Okada et al. (2015; 2018) that included hundred of millions of nodes for the whole-heart and human torso finite element model to generate virtual ECG. Applying simulation protocol as in Section 2.2 to the whole-heart simulations may be impractical because one whole-heart simulation may take much longer than a single-cell simulation depending on the number of nodes and elements within the finite element utilized in the model. However, multi-cell models such as 1D fiber and 2D tissue models may be feasible to combine with multi-drug and inter-individual variability *in silico* assessment as they consist of much smaller number of nodes compared to 3D heart simulations.

In conclusion, combining an electrophysiological model with optimized machine learning algorithms predicts drug-induced TdP risk accurately. The findings of this study provide valuable insights into developing more robust and comprehensive approaches to assess cardiotoxicity during drug development. Further refinement and validation of these models could greatly benefit the pharmaceutical industry by enabling early identification of potential drug-induced TdP risk.

## Data Availability

The raw data supporting the conclusion of this article will be made available by the authors, without undue reservation.

## References

[B1] Al-Faiz M. Z. Ibrahim A. A. Hadi S. M. (2018). The effect of z-score standardization on binary input due the speed of learning in back-propagation neural network. Iraqi J. Inf. Commun. Technol. 1, 42–48. 10.31987/ijict.1.3.41

[B2] Arif Ali Z. Abduljabbar H. Tahir A. Bibo Sallow A. Almufti S. M. (2023). eXtreme gradient boosting algorithm with machine learning: A review. Acad. J. Nawroz Univ. 12 (2), 320–334. 10.25007/ajnu.v12n2a1612

[B3] Belete D. M. Huchaiah M. D. (2022). Grid search in hyperparameter optimization of machine learning models for prediction of HIV/AIDS test results. Int. J. Comput. Appl. 44 (9), 875–886. 10.1080/1206212X.2021.1974663

[B4] Belgiu M. Drăguţ L. (2016). Random forest in remote sensing: A review of applications and future directions. ISPRS J. Photogrammetry Remote Sens. 114, 24–31. 10.1016/j.isprsjprs.2016.01.011

[B62] Cavero I. Crumb W. (2005). ICH S7B draft guideline on the non-clinical strategy for testing delayed cardiac repolarisation risk of drugs: a critical analysis. In Expert Opinion on Drug Safety 4 (3), 509–530. 10.1517/14740338.4.3.509 15934857

[B5] Chang K. C. Dutta S. Mirams G. R. Beattie K. A. Sheng J. Tran P. N. (2017a). Uncertainty quantification reveals the importance of data variability and experimental design considerations for *in silico* proarrhythmia risk assessment. Front. Physiology 8, 917. 10.3389/fphys.2017.00917 PMC5702340 29209226

[B6] Chang K. C. Dutta S. Mirams G. R. Beattie K. A. Sheng J. Tran P. N. (2017b). Uncertainty quantification reveals the importance of data variability and experimental design considerations for *in silico* proarrhythmia risk assessment. Front. Physiology 8, 917. 10.3389/fphys.2017.00917 PMC5702340 29209226

[B7] Chen T. Guestrin C. (2016). XGBoost: A scalable tree boosting system. arXiv. 10.1145/2939672.2939785

[B8] Colatsky T. Fermini B. Gintant G. Pierson J. B. Sager P. Sekino Y. (2016). The comprehensive *in vitro* proarrhythmia assay (CiPA) initiative — update on progress. J. Pharmacol. Toxicol. Methods 81, 15–20. 10.1016/j.vascn.2016.06.002 27282641

[B9] Crumb W. J. Vicente J. Johannesen L. Strauss D. G. (2016). An evaluation of 30 clinical drugs against the comprehensive *in vitro* proarrhythmia assay (CiPA) proposed ion channel panel. J. Pharmacol. Toxicol. Methods 81, 251–262. 10.1016/j.vascn.2016.03.009 27060526

[B10] Cunningham S. Ridley H. Weinel J. Picking R. (2021). Supervised machine learning for audio emotion recognition: enhancing film sound design using audio features, regression models and artificial neural networks. Personal Ubiquitous Comput. 25 (4), 637–650. 10.1007/s00779-020-01389-0

[B11] Dutta S. Chang K. C. Beattie K. A. Sheng J. Tran P. N. Wu W. W. (2017). Optimization of an *in silico* cardiac cell model for proarrhythmia risk assessment. Front. Physiology 8, 616. 10.3389/fphys.2017.00616 PMC5572155 28878692

[B12] Elgeldawi E. Sayed A. Galal A. R. Zaki A. M. (2021). Hyperparameter tuning for machine learning algorithms used for Arabic sentiment analysis. Informatics 8 (4), 79. 10.3390/informatics8040079

[B13] EMEA (2006). ICH topic S 7 B: the nonclinical evaluation of the potential for delayed ventricular repolarization (QT interval prolongation) by human pharmaceuticals. London: European Medicines Agency, November, 1–9.

[B14] Fuadah Y. N. Pramudito M. A. Lim K. M. (2023). An optimal approach for heart sound classification using grid search in hyperparameter optimization of machine learning. Bioengineering 10 (1), 45. 10.3390/bioengineering10010045 PMC9854602 36671616

[B15] FDA (2005). Guidance for industry interval prolongation and guidance for industry. E14 clinical evaluation of QT/QTc interval prolongation and proarrythmic potential for non-antiarrhythmic drugs. U.S. department of health and human services food and drug administration center for drug evaluation and research (CDER) center for biologics evaluation and research (CBER). Rockville: Food and Drug Administration, 1–16.

[B16] Frommeyer G. Eckardt L. (2016). Drug-induced proarrhythmia: risk factors and electrophysiological mechanisms. Nat. Rev. Cardiol. 13 (1), 36–47. 10.1038/nrcardio.2015.110 26194552

[B17] Gintant G. A. (2008). Preclinical torsades-de-pointes screens: advantages and limitations of surrogate and direct approaches in evaluating proarrhythmic risk. Pharmacol. Ther. 119 (2), 199–209. 10.1016/j.pharmthera.2008.04.010 18621077

[B18] Gressling T. (2021). “84 Automated machine learning,” in Artificial intelligence, big data, chemometrics and quantum computing with jupyter (De Gruyter: The Springer Series on Challenges in MachineLearning), 409–411. 10.1515/9783110629453-084

[B19] Hamzah A. S. Mohamed A. (2020). Classification of white rice grain quality using ann: A review. IAES Int. J. Artif. Intell. 9 (4), 600–608. 10.11591/ijai.v9.i4.pp600-608

[B20] Hendrawan I. R. Utami E. Hartanto A. D. (2022). Comparison of naïve bayes algorithm and XGBoost on local product review text classification. Edumatic J. Pendidik. Inform. 6 (1), 143–149. 10.29408/edumatic.v6i1.5613

[B21] Hill A. V. (1910). The heat produced in contracture and muscular tone. J. Physiology 40, 389–403. 10.1113/jphysiol.1910.sp001377 PMC1533709 16993015

[B22] Hwang M. Han S. Park M. C. Leem C. H. Shim E. B. Yim D. S. (2019). Three-dimensional heart model-based screening of proarrhythmic potential by *in silico* simulation of action potential and electrocardiograms. Front. Physiology 10, 1139. 10.3389/fphys.2019.01139 PMC6738014 31551815

[B23] Hwang M. Lim C. H. Leem C. H. Shim E. B. (2020). *In silico* models for evaluating proarrhythmic risk of drugs. Apl. Bioeng. 4 (2), 021502. 10.1063/1.5132618 32548538 PMC7274812

[B24] Ibrahem Ahmed Osman A. Najah Ahmed A. Chow M. F. Feng Huang Y. El-Shafie A. (2021). Extreme gradient boosting (Xgboost) model to predict the groundwater levels in Selangor Malaysia. Ain Shams Eng. J. 12 (2), 1545–1556. 10.1016/j.asej.2020.11.011

[B25] Jeong D. U. Nurul Qashri Mahardika T. Marcellinus A. Lim K. M. (2022). qInward variability-based *in-silico* proarrhythmic risk assessment of drugs using deep learning model. Front. Physiology 13, 1080190. 10.3389/fphys.2022.1080190 PMC9794579 36589462

[B26] Kubo T. Ashihara T. Tsubouchi T. Horie M. (2017). Significance of integrated *in silico* transmural ventricular wedge preparation models of human non-failing and failing hearts for safety evaluation of drug candidates. J. Pharmacol. Toxicol. Methods 83, 30–41. 10.1016/j.vascn.2016.08.007 27546811

[B27] Kun-Hee Kim K.-S. Lee H.-A. Han S.-H. Yim D.-S. (2018). Integrated *in vivo* cardiac safety evaluation using systemic pharmacology technique, 25–32. 10.23032/jaae.2018.12.1.002

[B28] Lancaster M. C. Sobie E. A. (2016). Improved prediction of drug-induced torsades de Pointes through simulations of dynamics and machine learning algorithms. Clin. Pharmacol. Ther. 100, 371–379. 10.1002/cpt.367 26950176 PMC6375298

[B29] Li Z. Ridder B. J. Han X. Wu W. W. Sheng J. Tran P. N. (2019). Assessment of an in silico mechanistic model for proarrhythmia risk prediction under the CiPA initiative. Clin. Pharmacol. Ther. 105 (2), 466–475. 10.1002/cpt.1184 30151907 PMC6492074

[B30] Llopis-Lorente J. Gomis-Tena J. Cano J. Romero L. Saiz J. Trenor B. (2020). *In silico* classifiers for the assessment of drug proarrhythmicity. J. Chem. Inf. Model. 60 (10), 5172–5187. 10.1021/acs.jcim.0c00201 32786710

[B31] Lundberg S. M. Erion G. Chen H. DeGrave A. Prutkin J. M. Nair B. (2020). From local explanations to global understanding with explainable AI for trees. Nat. Mach. Intell. 2 (1), 56–67. 10.1038/s42256-019-0138-9 32607472 PMC7326367

[B32] Luo C. Wang K. Zhang H. (2017a). Effects of amiodarone on short QT syndrome variant 3 in human ventricles: A simulation study. Biomed. Eng. Online 16 (1), 69. 10.1186/s12938-017-0369-0 28592292 PMC5463381

[B33] Luo C. Wang K. Zhang H. (2017b). *In silico* assessment of the effects of quinidine, disopyramide and E-4031 on short QT syndrome variant 1 in the human ventricles. PLoS ONE 12 (6), e0179515. 10.1371/journal.pone.0179515 28632743 PMC5478111

[B34] Mirams G. R. Cui Y. Sher A. Fink M. Cooper J. Heath B. M. (2011). Simulation of multiple ion channel block provides improved early prediction of compounds' clinical torsadogenic risk. Cardiovasc. Res. 91 (1), 53–61. 10.1093/cvr/cvr044 21300721 PMC3112019

[B35] Montomoli J. Romeo L. Moccia S. Bernardini M. Migliorelli L. Berardini D. (2021). Machine learning using the extreme gradient boosting (XGBoost) algorithm predicts 5-day delta of SOFA score at ICU admission in COVID-19 patients. J. Intensive Med. 1 (2), 110–116. 10.1016/j.jointm.2021.09.002 36785563 PMC8531027

[B36] Obiol-Pardo C. Gomis-Tena J. Sanz F. Saiz J. Pastor M. (2011). A multiscale simulation system for the prediction of drug-induced cardiotoxicity. J. Chem. Inf. Model. 51 (2), 483–492. 10.1021/ci100423z 21250697

[B37] O’Hara T. Virág L. Varró A. Rudy Y. (2011). Simulation of the undiseased human cardiac ventricular action potential: model formulation and experimental validation. PLoS Comput. Biol. 7 (5), e1002061. 10.1371/journal.pcbi.1002061 21637795 PMC3102752

[B38] Okada J. I. Yoshinaga T. Kurokawa J. Washio T. Furukawa T. Sawada K. (2018). Arrhythmic hazard map for a 3D whole-ventricle model under multiple ion channel block. Br. J. Pharmacol. 175 (17), 3435–3452. 10.1111/bph.14357 29745425 PMC6086978

[B39] Okada J. I. Yoshinaga T. Kurokawa J. Washio T. Furukawa T. Sawada K. (2015). Screening system for drug-induced arrhythmogenic risk combining a patch clamp and heart simulator. Sci. Adv. 1 (4), e1400142. 10.1126/sciadv.1400142 26601174 PMC4640654

[B40] Pantic I. Paunovic J. Cumic J. Valjarevic S. Petroianu G. A. Corridon P. R. (2023). Artificial neural networks in contemporary toxicology research. Chemico-Biological Interact. 369, 110269. 10.1016/j.cbi.2022.110269 36402212

[B41] Parikh J. Gurev V. Rice J. J. (2017). Novel two-step classifier for Torsades de Pointes risk stratification from direct features. Front. Pharmacol. 8, 816. 10.3389/fphar.2017.00816 29184497 PMC5694470

[B42] Parmar A. Katariya R. Patel V. (2019). “A review on random forest: an ensemble classifier,” in International conference on intelligent data communication technologies and internet of things (ICICI) 2018, 758–763. 10.1007/978-3-030-03146-6_86

[B43] Passini E. Britton O. J. Lu H. R. Rohrbacher J. Hermans A. N. Gallacher D. J. (2017). Human *in silico* drug trials demonstrate higher accuracy than animal models in predicting clinical pro-arrhythmic cardiotoxicity. Front. Physiology 8, 668. 10.3389/fphys.2017.00668 PMC5601077 28955244

[B44] Polak S. Romero K. Berg A. Patel N. Jamei M. Hermann D. (2018). Quantitative approach for cardiac risk assessment and interpretation in tuberculosis drug development. J. Pharmacokinet. Pharmacodynamics 45 (3), 457–467. 10.1007/s10928-018-9580-2 PMC5953981 29520534

[B45] Qauli A. I. Yoo Y. Marcellinus A. Lim K. M. (2022). Verification of the efficacy of mexiletine treatment for the A1656D mutation on downgrading reentrant tachycardia using a 3D cardiac electrophysiological model. Bioengineering 9 (10), 531. 10.3390/bioengineering9100531 36290499 PMC9598628

[B46] Raju V. N. G. Lakshmi K. P. Jain V. M. Kalidindi A. Padma V. (2020). “Study the influence of normalization/transformation process on the accuracy of supervised classification,” in 2020 Third International Conference on Smart Systems and Inventive Technology (ICSSIT), Tirunelveli, India, 20-22 August 2020 (IEEE), 729–735. 10.1109/ICSSIT48917.2020.9214160

[B47] Romero L. Cano J. Gomis-Tena J. Trenor B. Sanz F. Pastor M. (2018). *In silico* QT and APD prolongation assay for early screening of drug-induced proarrhythmic risk. J. Chem. Inf. Model. 58 (4), 867–878. 10.1021/acs.jcim.7b00440 29547274

[B48] Sha’abani M. N. A. H. Fuad N. Jamal N. Ismail M. F. (2020). “kNN and SVM classification for eeg: A review,” in InECCE2019 (Singapore: Springer), 555–565.

[B49] Shanbehzadeh M. Nopour R. Kazemi-Arpanahi H. (2022). Design of an artificial neural network to predict mortality among COVID-19 patients. Inf. Med. Unlocked 31, 100983. 10.1016/j.imu.2022.100983 PMC9148440 35664686

[B50] Sharma D. K. Chatterjee M. Kaur G. Vavilala S. (2022). “3 - deep learning applications for disease diagnosis,” in Deep learning for medical applications with unique data (Academic Press), 31–51. 10.1016/B978-0-12-824145-5.00005-8

[B51] Stavropoulos G. van Voorstenbosch R. van Schooten F. J. Smolinska A. (2020). “Random forest and ensemble methods,” in Comprehensive chemometrics (Elsevier), 661–672. 10.1016/b978-0-12-409547-2.14589-5

[B52] Štrumbelj E. Kononenko I. (2014). Explaining prediction models and individual predictions with feature contributions. Knowl. Inf. Syst. 41 (3), 647–665. 10.1007/s10115-013-0679-x

[B53] Suhail Y. Upadhyay M. Chhibber A. Kshitiz S. (2020). Machine learning for the diagnosis of orthodontic extractions: A computational analysis using ensemble learning. Bioengineering 7 (2), 55–13. 10.3390/bioengineering7020055 32545428 PMC7355468

[B54] Tarwidi D. Pudjaprasetya S. R. Adytia D. Apri M. (2023). An optimized XGBoost-based machine learning method for predicting wave run-up on a sloping beach. MethodsX 10, 102119. 10.1016/j.mex.2023.102119 37007622 PMC10064230

[B55] Tomek J. Bueno-Orovio A. Passini E. Zhou X. Minchole A. Britton O. (2019). Development, calibration, and validation of a novel human ventricular myocyte model in health, disease, and drug block. ELife 8, e48890. 10.7554/eLife.48890 31868580 PMC6970534

[B56] Tuan Hoang A. Nižetić S. Chyuan Ong H. Tarelko W. Viet Pham V. Hieu Le T. (2021). A review on application of artificial neural network (ANN) for performance and emission characteristics of diesel engine fueled with biodiesel-based fuels. Sustain. Energy Technol. Assessments 47, 101416. 10.1016/j.seta.2021.101416

[B57] Uddin S. Haque I. Lu H. Moni M. A. Gide E. (2022). Comparative performance analysis of K-nearest neighbour (KNN) algorithm and its different variants for disease prediction. Sci. Rep. 12 (1), 6256. 10.1038/s41598-022-10358-x 35428863 PMC9012855

[B58] Xia Y. (2020). Correlation and association analyses in microbiome study integrating multiomics in health and disease. Prog. Mol. Biol. Transl. Sci. 171, 309–491. 10.1016/bs.pmbts.2020.04.003 32475527

[B59] Yoo Y. Marcellinus A. Jeong D. U. Kim K. S. Lim K. M. (2021). Assessment of drug proarrhythmicity using artificial neural networks with *in silico* deterministic model outputs. Front. Physiology 12, 761691. 10.3389/fphys.2021.761691 PMC8703011 34955882

[B60] Zampieri M. Soranzo N. Bianchini D. Altafini C. (2008). Origin of co-expression patterns in E.coli and S.cerevisiae emerging from reverse engineering algorithms. PLoS ONE 3 (8), e2981. 10.1371/journal.pone.0002981 18714358 PMC2500178

[B61] Zhou X. Qu Y. Passini E. Bueno-Orovio A. Liu Y. Vargas H. M. (2020). Blinded *in silico* drug trial reveals the minimum set of ion channels for torsades de pointes risk assessment. Front. Pharmacol. 10, 1643. 10.3389/fphar.2019.01643 32082155 PMC7003137

