# Supplementary material for: Machine learning approach to evaluate TdP risk of drugs using cardiac electrophysiological model including inter-individual variability
Source: Front Physiol. 2023 Oct 4;14:1266084. doi: 10.3389/fphys.2023.1266084 (PMC10584148; doi:10.3389/fphys.2023.1266084)
Supplement: Supplementary file 1 [file DataSheet1.docx]

Supplementary Material

Machine Learning Approach to Evaluate TdP Risk of Drugs Using Cardiac Electrophysiological Model Including Inter-Individual Variability

# Design of the Virtual Population of Human Ventricular Cell Models

We generated a virtual population of human ventricular cell models based on an inter-individual variation of parameters using a modified O'Hara Rudy model. The virtual population follows the control population of a human ventricular model that was experimentally calibrated in previous studies (Britton et al., 2013; Muszkiewicz et al., 2016; Passini et al., 2017, 2019). Each of the nine main ionic conductances was varied randomly using uniform distribution under the range of variations, as shown in Table S1.

We built a 10,000 initial population of human ventricular cell model, and a healthy control population of 1,151 individuals was selected based on healthy AP criteria (Britton et al., 2017; O’Hara et al., 2011; Passini et al., 2017) and Ca^2+^ transient (Coppini et al., 2013; Passini et al., 2016) as shown in Table S2. The corresponding biomarkers were the intersecting biomarkers used for evaluation of TdP risk of drugs as in Dutta et al. (2017) and the biomarkers used in Passini et al. (2019).

Table S1. Variability range percentage for each conductance in designing the control population human ventricular cell models based on previous studies (Britton et al., 2013; Passini et al., 2017).

| **Model Parameter** | **Variability Range** |
| --- | --- |
| $G_{\mathrm{Na}}$ | $\left[ 30-200 \right]\%$ |
| $G_{\mathrm{Nal}}$ | $\left[ 100-200 \right]\%$ |
| $G_{\mathrm{to}}$ | $\left[ 0-200 \right]\%$ |
| $G_{\mathrm{Kr}}$ | $\left[ 45-100 \right]\%$ |
| $G_{\mathrm{Ks}}$ | $\left[ 0-100 \right]\%$ |
| $G_{K1}$ | $\left[ 30-200 \right]\%$ |
| $G_{\mathrm{NCX}}$ | $\left[ 100-200 \right]\%$ |
| $G_{\mathrm{NaK}}$ | $\left[ 30-100 \right]\%$ |
| $G_{\mathrm{CaL}}$ | $\left[ 100-200 \right]\%$ |

Table S2. Healthy AP criteria based on previous studies (Britton et al., 2017; O’Hara et al., 2011; Passini et al., 2017) and Ca^2+^ transient based on previous studies (Coppini et al., 2013; Passini et al., 2016).

| **AP biomarker** | **Min value** | **Max Value** |
| --- | --- | --- |
| $\mathrm{APD}_{50}$ | 110 ms | 350 ms |
| $\mathrm{APD}_{90}$ | 180 ms | 440 ms |
| $\left( \frac{\mathrm{dV}}{\mathrm{dt}} \right)_{\mathrm{Max}}$ | 100 V/s | 1000 V/s |
| $\mathrm{Vm}_{\mathrm{peak}}$ | 10 mV | 55 mV |
| $\mathrm{Vm}_{\mathrm{resting}}$ | -95 mV | -80 mV |
| $\mathrm{CaD}_{50}$ | 120 ms | 420 ms |
| $\mathrm{CaD}_{90}$ | 220 ms | 785 ms |

1. **Feature correlation**


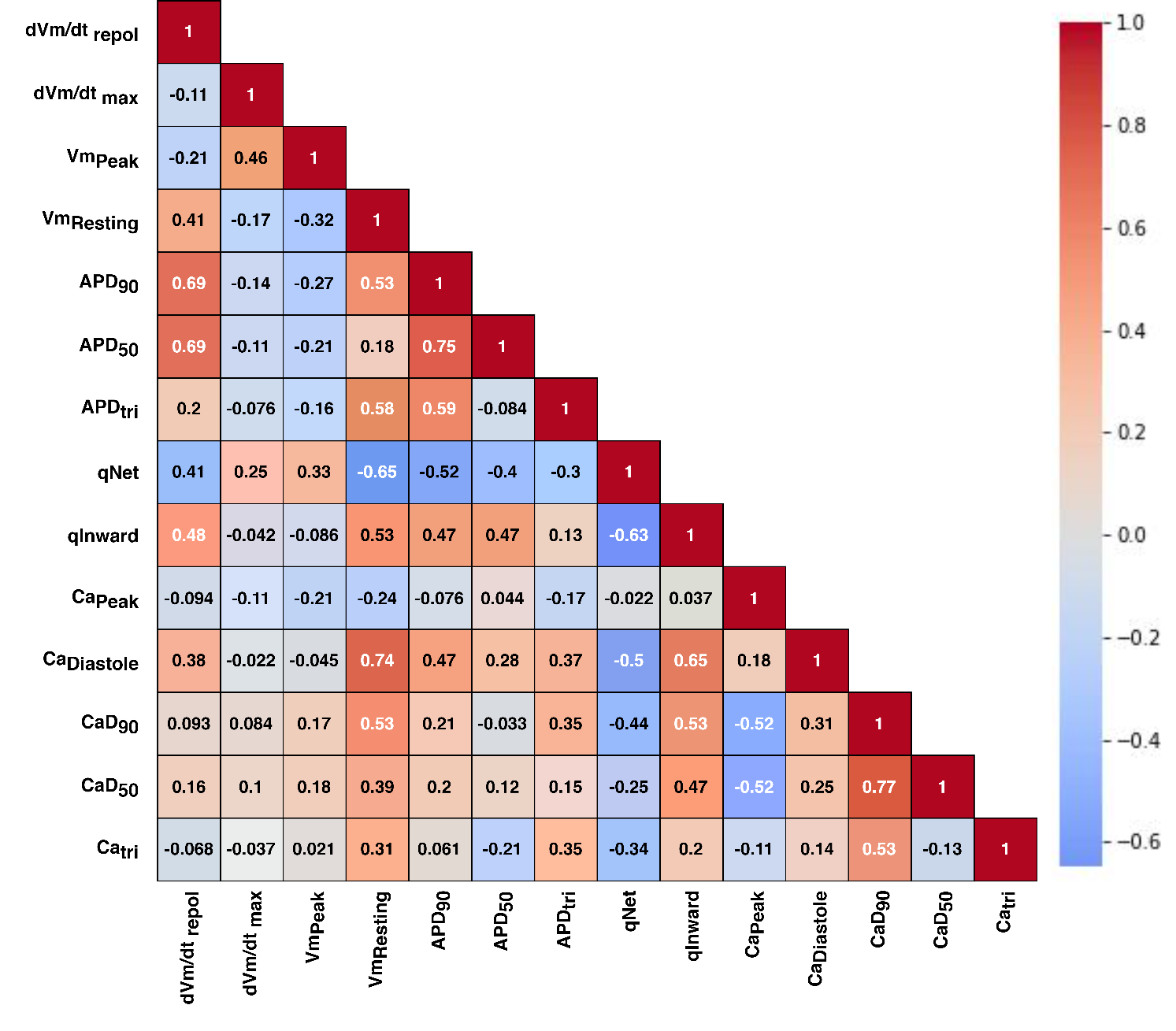


Figure S1. Correlation heatmap of 14 *in silico* features.

1. **Categorization of TdP risk of drugs into high, intermediate, and low-risk groups according to CIPA**

In the previous study by Passini et al. (2017) they provided a dataset consisting of 39 drugs with high TdP risk, 28 drugs with no/low TdP risk, and 12 drugs with possible/intermediate risk. For binary classification, they only used high TdP risk and no TdP risk groups. In this study, we also tried multi-class classification of TdP risk group. However, the number of intermediate-risk drugs in the dataset provided by Passini et al. (2017) was limited. Therefore, we distributed some drugs from the high-risk group into the intermediate-risk group according to the CIPA categorization. Several drugs from the high-risk group in Passini et al. (2017) including astemizole, chloropromazine I, chloropromazine II, cisapride I, cisapride II, droperidol, pimozide, terfenadine I, and terfenadine II are adjusted into intermediate risk group (since these drugs according to the CIPA belong to intermediate risk group). Table S3 shows the list of drugs for high, intermediate, and no TdP risk groups used in this study for 3 class TdP risk of drugs classification.

Table S3. The list of train and test drugs with EFPTCmax value for high, intermediate, and no TdP risk groups.

| **Proarrhythmic risk level** | **Train drugs** | | **Test drugs** | |
| --- | --- | --- | --- | --- |
|  | **Name** | **EFTPCmax (µM)** | **Name** | **EFTPCmax(µM)** |
| **High TdP-risk** | Amiodarone I | 0.155 | Moxifloxacin I | 10.96 |
|  | Amiodarone II | 0.155 | Moxifloxacin II | 10.96 |
|  | Bepridil I | 0.035 | Moxifloxacin III | 10.96 |
|  | Bepridil II | 0.035 | Procainamide | 54.18 |
|  | Bepridil III | 0.035 | Quinidine | 3.237 |
|  | Cilostazol | 0.128 | Quinidine1 | 3.237 |
|  | Disopyramide | 0.742 | Sotalol I | 14.69 |
|  | Dofetilide I | 0.0021 | Sotalol II | 14.69 |
|  | Dofetilide II | 0.0021 | Sparfloxacin I | 1.766 |
|  | Dofetilide III | 0.0021 | Sparfloxacin II | 1.766 |
|  | Donepezil | 0.007 | Terodiline | 0.145 |
|  | Flecainide I | 0.752 | Thioridazine | 0.98 |
|  | Flecainide II | 0.752 |  |  |
|  | Flecainide III | 0.752 |  |  |
|  | Halofantrine | 0.172 |  |  |
|  | Haloperidol | 0.004 |  |  |
|  | Ibutilide | 0.14 |  |  |
|  | Methadone | 0.507 |  |  |
| **Intermediate TdP-risk** | Astemizole | 0.0003 | Pimozide | 0.0005 |
|  | Chlorpromazine I | 0.038 | Sertindole I | 0.002 |
|  | Chlorpromazine II | 0.038 | Sertindole II | 0.002 |
|  | Cisapride I | 0.003 | Solifenacin | 0.003 |
|  | Cisapride II | 0.003 | Sunitinib | 0.013 |
|  | Clozapine | 0.071 | Terfenadine I | 0.009 |
|  | Dasatinib | 0.041 | Terfenadine II | 0.009 |
|  | Droperidol | 0.016 |  |  |
|  | Nilotinib I | 0.172 |  |  |
|  | Nilotinib II | 0.172 |  |  |
|  | Paliperidone | 0.069 |  |  |
|  | Risperidone | 0.002 |  |  |
|  | Saquinavir | 0.4172 |  |  |
|  | Saquinavir I | 0.4172 |  |  |
| **Low TdP-risk** | BaCl2 | 1 | Nisoldipine | 0.0001 |
|  | Ceftriaxone | 23.17 | Nitrendipine | 0.003 |
|  | Diazepam | 0.029 | Pentobarbital | 5.171 |
|  | Diltiazem I | 0.1275 | Phenytoin | 4.36 |
|  | Diltiazem II | 0.1275 | Primidone | 20.6 |
|  | Duloxetine | 0.016 | Piperacillin | 114 |
|  | Lamivudine | 19.54 | Raltegravir | 7 |
|  | Lidocaine I | 2.6 | Ribavirin | 27.88 |
|  | Lidocaine II | 2.6 | Sitagliptin | 0.442 |
|  | Linezolid | 59.11 | Telbivudine | 19.72 |
|  | Loratadine | 0.0004 |  |  |
|  | Mexiletine I | 2.5 |  |  |
|  | Mexiletine II | 2.5 |  |  |
|  | Mibefradil I | 0.012 |  |  |
|  | Mibefradil II | 0.012 |  |  |
|  | Mitoxantrone | 0.225 |  |  |
|  | Nifedipine | 0.008 |  |  |
|  | Nimodipine | 0.001 |  |  |

1. **The advantages and disadvantages of ANN, Random Forest, XGBoost, and K-NN**

In this study, we evaluated the performance of 4 machine learning models in predicting the TdP risk of drugs: ANN, Random Forest, XGBoost, and K-NN. There are several advantages and disadvantages of those machine learning, as shown in Table 4. These advantages and disadvantages have correlated with the performance result provided by each machine learning model.

Table S4. The advantages and disadvantages of ANN, Random Forest, XGBoost, and K-NN.

| **No** | **Classifier Models** | **Advantages** | **Disadvantages** |
| --- | --- | --- | --- |
| 1 | **KNN** | **Simplicity:** KNN is easy to understand and implement, making it a good choice for binary classification task such as high TdP and no TdP risk classification. | **Computational Complexity:** Prediction with KNN required high computation time for a large dataset, as it requires calculating distances to all data points. |
|  |  | **Non-parametric:** KNN makes no assumptions about the underlying data distribution, allowing it to handle diverse datasets. | **Selecting K-value:** Selecting the optimal value of 'k' (number of neighbors) can impact the model's performance and required high computational time. |
|  |  | **Adaptability:** KNN can be used for both classification and regression tasks. | **Sensitive to Noise and Outliers:** KNN is sensitive to noisy data and outliers, which can lead to inaccurate predictions |
|  |  | **Local Patterns:** KNN captures local patterns and can be effective in cases where data clusters are present. | **Curse of Dimensionality:** KNN's performance can degrade in high-dimensional spaces due to the increased sparsity of data . |
| 2 | **XGBoost** | **Ensemble Learning:** XGBoost employs a boosting technique to sequentially improve model performance by correcting errors. | **Complexity:** Setting hyperparameters in XGBoost requires careful tuning to achieve optimal results |
|  |  | **High Performance:** It often outperforms other algorithms due to its ability to handle complex relationships and interactions. | **Resource Intensive:** XGBoost can be computationally demanding and memory-intensive for large datasets. |
|  |  | **Regularization:** XGBoost includes regularization terms to prevent overfitting and improve generalization ability of the model. | **Black Box:** Interpreting the model's decisions can be challenging due to its complexity, similar to other ensemble methods. |
|  |  | **Feature Importance:** It provides feature importance scores that aid in understanding the contribution of variables. | **Potential for Overfitting:** While regularization helps, improper parameter tuning can still lead to overfitting. |
| 3 | **RF** | **Ensemble Learning:** RF combines multiple decision trees to improve overall prediction accuracy and reduce overfitting. | **Memory and Computational Requirements:** Training multiple decision trees can be resource-intensive for large datasets. |
|  |  | **Feature Importance:** RF provides insights into feature importance, helping to identify relevant predictors | **Bias towards Dominant Classes:** RF can be biased towards predicting dominant classes for the imbalanced dataset. |
|  |  | **Handles Various Data Types:** RF can handle both categorical and numerical features without much pre-processing. | **Not Suitable for High-Dimensional Data:** In cases of high-dimensional data, RF might struggle to capture complex interactions. |
|  |  | **Robustness:** RF is less sensitive to outliers and noise in the data. |  |
| 4 | **ANN** | **Non-linearity:** ANNs can model complex relationships and capture non-linear patterns in data. | **Complexity:** Designing and training ANNs can be intricate, requiring careful tuning of hyperparameters and architecture. |
|  |  | **Feature Learning:** ANN can automatically extract the relevant features from raw data. | **Data Intensity:** ANNs often require a substantial amount of labeled data for training, making them less suitable for small datasets. |
|  |  | **Flexibility:** ANN adaptable to various problem domains through adjustments in architecture and hyperparameters. | **Black Box:** Interpreting the decisions made by ANNs can be challenging due to their complex structure. |
|  |  | **Scalability**: Deep neural networks showed good performance in tasks involving large dataset.  **Generalization**: ANN has a good generalization ability to classify the unseen data.  **Robustness**: ANN can be designed to be robust against various types of noise and outliers. | **Overfitting:** ANNs are prone to overfitting, especially when the model is too large or the training data is limited . |

**References**

Britton, O. J., Bueno-Orovio, A., Van Ammel, K., Lu, H. R., Towart, R., Gallacher, D. J., & Rodriguez, B. (2013). Experimentally calibrated population of models predicts and explains intersubject variability in cardiac cellular electrophysiology. *Proceedings of the National Academy of Sciences of the United States of America*, *110*(23). https://doi.org/10.1073/pnas.1304382110

Britton, O. J., Bueno-Orovio, A., Virág, L., Varró, A., & Rodriguez, B. (2017). The electrogenic Na+/K+ pump is a key determinant of repolarization abnormality susceptibility in human ventricular cardiomyocytes: A population-based simulation study. *Frontiers in Physiology*, *8*(MAY). https://doi.org/10.3389/fphys.2017.00278

Coppini, R., Ferrantini, C., Yao, L., Fan, P., Del Lungo, M., Stillitano, F., Sartiani, L., Tosi, B., Suffredini, S., Tesi, C., Yacoub, M., Olivotto, I., Belardinelli, L., Poggesi, C., Cerbai, E., & Mugelli, A. (2013). Late sodium current inhibition reverses electromechanical dysfunction in human hypertrophic cardiomyopathy. *Circulation*, *127*(5), 575–584. https://doi.org/10.1161/CIRCULATIONAHA.112.134932

Dutta, S., Chang, K. C., Beattie, K. A., Sheng, J., Tran, P. N., Wu, W. W., Wu, M., Strauss, D. G., Colatsky, T., & Li, Z. (2017). Optimization of an in silico cardiac cell model for proarrhythmia risk assessment. *Frontiers in Physiology*, *8*(AUG). https://doi.org/10.3389/fphys.2017.00616

Muszkiewicz, A., Britton, O. J., Gemmell, P., Passini, E., Sánchez, C., Zhou, X., Carusi, A., Quinn, T. A., Burrage, K., Bueno-Orovio, A., & Rodriguez, B. (2016). Variability in cardiac electrophysiology: Using experimentally-calibrated populations of models to move beyond the single virtual physiological human paradigm. *Progress in Biophysics and Molecular Biology*, *120*(1–3), 115–127. https://doi.org/10.1016/j.pbiomolbio.2015.12.002

O’Hara, T., Virág, L., Varró, A., & Rudy, Y. (2011). Simulation of the undiseased human cardiac ventricular action potential: Model formulation and experimental validation. *PLoS Computational Biology*, *7*(5). https://doi.org/10.1371/journal.pcbi.1002061

Passini, E., Britton, O. J., Lu, H. R., Rohrbacher, J., Hermans, A. N., Gallacher, D. J., Greig, R. J. H., Bueno-Orovio, A., & Rodriguez, B. (2017). Human in silico drug trials demonstrate higher accuracy than animal models in predicting clinical pro-arrhythmic cardiotoxicity. *Frontiers in Physiology*, *8*(SEP). https://doi.org/10.3389/fphys.2017.00668

Passini, E., Mincholé, A., Coppini, R., Cerbai, E., Rodriguez, B., Severi, S., & Bueno-Orovio, A. (2016). Mechanisms of pro-arrhythmic abnormalities in ventricular repolarisation and anti-arrhythmic therapies in human hypertrophic cardiomyopathy. *Journal of Molecular and Cellular Cardiology*, *96*, 72–81. https://doi.org/10.1016/j.yjmcc.2015.09.003

Passini, E., Trovato, C., Morissette, P., Sannajust, F., Bueno-Orovio, A., & Rodriguez, B. (2019). Drug-induced shortening of the electromechanical window is an effective biomarker for in silico prediction of clinical risk of arrhythmias. *British Journal of Pharmacology*, *176*(19), 3819–3833. https://doi.org/10.1111/bph.14786
